# Effectiveness of Progressive Resistive Exercise (PRE) in the context of HIV: systematic review and meta-analysis using the Cochrane Collaboration protocol

**DOI:** 10.1186/s12879-017-2342-8

**Published:** 2017-04-12

**Authors:** Kelly K. O’Brien, Anne-Marie Tynan, Stephanie A. Nixon, Richard H. Glazier

**Affiliations:** 1 Department of Physical Therapy, University of Toronto, 500 University Avenue, Room 160, Toronto, ON Canada; 2 Rehabilitation Sciences Institute (RSI), University of Toronto, 500 University Avenue, Room 160, Toronto, ON Canada; 3 Institute of Health Policy, Management and Evaluation (IHPME), University of Toronto, Toronto, ON Canada; 4 Centre for Urban Health Solutions (CUHS), Li Ka Shing Knowledge Institute, 30 Bond Street, St. Michael’s Hospital, Toronto, ON Canada; 5 Institute for Clinical Evaluative Sciences, G1 06 2075 Bayview Ave., Toronto, ON Canada; 6 Department of Family and Community Medicine, St. Michael’s Hospital, 30 Bond Street, Toronto, ON Canada; 7 Department of Family and Community Medicine, University of Toronto, 500 University Avenue, Toronto, ON Canada

**Keywords:** HIV/AIDS, Exercise, Resistive exercise, Strength training, Systematic review

## Abstract

**Background:**

HIV is increasingly considered a chronic illness. More individuals are living longer and aging with the health-related consequences associated with HIV and multi-morbidity. Exercise is a self-management approach that can promote health for people aging with HIV. We examined the safety and effectiveness of progressive resistive exercise (PRE) interventions on immunological, virological, cardiorespiratory, strength, weight, body composition, and psychological outcomes in adults living with HIV.

**Methods:**

We conducted a systematic review using the Cochrane Collaboration protocol. Searching databases up to April 2013, we included randomized controlled trials that compared PRE with no exercise or another intervention performed at least three times per week for at least four weeks with adults living with HIV. Two reviewers independently determined study eligibility. We extracted data from included studies and assessed risk of bias using the Cochrane Collaboration risk of bias tool. Meta-analyses were conducted using random effects models with Review Manager (RevMan) computer software.

**Results:**

Twenty studies met inclusion criteria (*n* = 764 participants at study completion); the majority of participants were men (77%) taking antiretroviral therapy (14/20 included studies). Exercise interventions included PRE alone (8 studies) or a combination of resistive and aerobic exercise (12 studies) ranging from 6 to 52 weeks in duration. Thirty-four meta-analyses were performed. Results demonstrated statistically significant improvements in cardiorespiratory status (maximum oxygen consumption, exercise time), strength (chest press, knee flexion), weight, and body composition (arm and thigh girth, leg muscle area) among exercisers versus non-exercisers. We found no significant differences in change in CD4 count and viral load. We were unable to perform meta-analyses for psychological outcomes however results from individual studies demonstrated improvements in health-related quality of life with exercisers compared with non-exercisers.

**Conclusions:**

Performing progressive resistive exercise (PRE) or a combination of resistive and aerobic exercise at least three times per week for at least six weeks is safe and can lead to improvements in cardiorespiratory fitness, strength, weight, and body composition for adults with HIV. Exercise may be considered a safe and beneficial for enhancing the health of medically stable adults aging with HIV.

**Electronic supplementary material:**

The online version of this article (doi:10.1186/s12879-017-2342-8) contains supplementary material, which is available to authorized users.

## Background

HIV is increasingly considered a chronic illness for people who have access to combination antiretroviral therapy. Many individuals are living longer and now aging with the health-related consequences of HIV and associated multi-morbidity [1–4]. These health consequences (known as disability) may include physical, cognitive, mental and emotional symptoms and impairments, difficulties with daily activities, challenges to social inclusion, and uncertainty or worrying about future health [5].

Exercise is a self-management strategy that can address disability and improve or sustain the health of people aging with HIV and multi-morbidity [6]. Exercise can improve strength, cardiovascular fitness, and quality of life outcomes in healthy populations [7, 8] and among people living with complex chronic illness [9]. Comparable benefits were found in an former version of this systematic review examining the effect of resistive exercise among adults living with HIV [10]. However, issues continue to emerge since the advent of combination antiretroviral therapy such as the risk of cardiovascular disease, diabetes, and changes in body composition for adults living with HIV [11]. A recent systematic review focused on aerobic exercise demonstrated benefits to cardiorespiratory fitness, strength, weight, body composition, and quality of life for adults with HIV [12]. Nonetheless, awareness of the benefits and risks of strength and resistance training, and optimal parameters for exercise for adults living with HIV continues to emerge. By better understanding the risks and benefits of exercise in the context of HIV, particularly the impact of resistance training, suitable exercise may be more widely adopted by people living with HIV; and healthcare providers may better promote safe and effective uptake of exercise in clinical practice.

The purpose of this study was to examine the safety and effectiveness of progressive resistive exercise (PRE) interventions on immunological, virological, cardiorespiratory, strength, weight, body composition, and psychological outcomes in adults living with HIV.

## Methods

We conducted a systematic review using the Cochrane Collaboration protocol [13].

### Inclusion criteria

We included randomized controlled trials (RCTs) that compared progressive resistance exercise (PRE) (or combinations of progressive resistance and aerobic exercise) with no PRE or another exercise or treatment intervention performed at least three times per week for at least four weeks [14]. Similar to our previous review, “we included studies of adults (18 years of age and older) living with HIV at all stages of infection with or without comorbidities” [12]. We defined PRE as any intervention that contained resistive exercise interventions performed at least three times per week for at least four weeks. PRE involved any activity containing resistive exercise that included but was not limited to weight strengthening, isotonic and isometric strengthening exercises. We included both supervised and un-supervised interventions [14].

### Outcomes

We assessed outcomes including “immunological (CD4 count, cells/mm^3^) and virological (viral load, log10 copies) outcomes. Cardiorespiratory measures included but were not limited to maximal oxygen consumption (VO2max), exercise time, maximum heart rate, and rate of perceived exertion. Strength measures included amount of weight able to resist in kilograms (1-repetition maximum) for major muscle groups. Weight and body composition measures included any outcome that contributes to the direct or indirect measurement of muscle, fat, bone or other tissues of the body. These included but were not limited to body weight, body mass index, lean body mass, girth, percent body fat, cross-sectional muscle area, and waist and hip circumference. Psychological measures included general measures of psychological status and health-related quality of life” [12].

### Search strategy

In the update of this systematic review, we searched databases from 2009 to April 19 2013 including “Medline, Cochrane Central Register of Controlled Trials, Cochrane Database of Systematic Reviews, Database of Abstracts of Reviews of Effects, PsycINFO, CINAHL, EMBASE, Web of Science: Science Citation Index, SPORTdiscus, Virology and AIDS Abstracts and LILACS. We also searched https://clinicaltrials.gov/ and reference lists from pertinent articles. All languages were included” [12]. See Additional file 1 for the MEDLINE search strategy that we modified for use with other databases.

### Selection of included studies

Abstracts yielded “from the search were reviewed independently by two reviewers (KKO and AMT)” [12]. We identified abstracts of studies that met the following criteria: a) participants were adults (18 years of age or older) living with HIV; and b) the study included a resistive exercise intervention performed at least three times per week, at least 20 min per session for at least four weeks; and c) included a randomized controlled comparison group.

“When one or both raters of the abstracts believed the study met eligibility criteria then full versions of the article were independently reviewed by the two reviewers to determine inclusion. In instances where there was a lack of agreement by the two reviewers, a third reviewer reviewed the full article to determine final inclusion” [12].

### Data extraction

Two reviewers (KKO, and/or AMT and SAN) independently extracted the data onto data extraction forms. “Data extracted included the study citation, study objectives, study design, length of study, time at which participants were assessed, inclusion and exclusion criteria for participants, characteristics of included participants (i.e., age, gender, stage of disease, comorbidity), description of intervention(s) (i.e., frequency, intensity, duration, type, level of supervision, location of intervention), types of outcome variables assessed and their values at baseline and study completion, and number of participants at baseline and study completion (including number of withdrawals) [12]. The reviewers met to achieve consensus regarding any difference in data interpretation or extraction from included studies that arose during the review process” [12]. Authors of included studies were contacted to obtain additional information or clarification if needed.

“Two authors assessed the risk of bias in the included studies using the Cochrane Collaboration tool for assessing risk of bias [15]. Potential biases may have included selection bias (random sequence generation and allocation concealment which may result in systematic differences in the comparison groups), performance bias (lack of blinding of participants and personnel which could lead to systematic differences in the care provided apart from the intervention being evaluated), detection bias (lack of blinding of outcome assessment that may result in systematic differences in outcome assessment), attrition bias (incomplete outcome data), and reporting bias (selective reporting of outcomes)” [12, 15].

We used the Grading of Recommendations Assessment, Development, and Evaluation (GRADE) method, to assess the overall quality of evidence for the main comparison of PRE or combined PRE and aerobic exercise versus no exercise with the following seven outcomes: viral load, VO2max, upper body strength, lower body strength, body weight, body mass index and fat mass [16]. These seven outcomes (also included in a summary of findings table) were chosen based on their perceived clinical importance and importance to adults living with HIV [17]. “We rated the quality of evidence for outcomes based on categories of very low, low, moderate and high [18]. We downgraded the evidence from high quality by one level for each of the following: attrition bias (where withdrawal rates were >15%), performance bias (when participants were not blinded to the intervention), detection bias (when assessors of outcomes were not blinded to group allocation), publication bias (when publication bias was suspected), and inconsistency (when moderate I^2^ ≥40% or substantial I^2^ > 75% heterogeneity exists)” [12, 19, 20].

We developed a summary of findings (SoF) table for the main comparison of PRE or combined PRE and aerobic exercise versus no exercise with the above seven outcomes. “The SoF table was developed to illustrate the confidence in the effect estimates (quality of evidence using the GRADE method) and magnitude of effect for the seven key outcomes” [12, 17].

### Analysis

“Outcomes were analyzed as continuous and dichotomous outcomes whenever possible. Meta-analyses were performed using the random-effects model for outcomes using Review Manager (RevMan) computer software whenever sufficient data were available, when similar or comparable outcome measures were used, and when participant comparison groups were similar” [12, 21].

“For continuous outcomes, the weighted mean difference (WMD) and 95% confidence intervals for the means were calculated whenever possible. For dichotomous outcomes, the odds ratio, absolute difference in odds, relative risk (RR), risk difference (RD), and the number needed to treat (NNT) and 95% confidence intervals for dichotomous outcomes whenever possible. A p value of less than 0.05 indicated statistical significance for overall effect” [12]. We performed subgroup analyses when possible to estimate whether PRE interventions were associated with differences among groups.

Similar to our previous systematic review, “we considered 50 cells/mm^3^ to indicate a clinically important change in CD4 count and 0.5 log10 copies to indicate a clinically important change in viral load. For cardiorespiratory outcomes, we considered 2 mL/kg/min to indicate a clinically important change in VO2max, 10 beats per minute to indicate a clinically important change in heart rate maximum (HRmax), and 5 min to indicate a clinically important change in exercise time. For strength outcomes we considered 5 kg to indicate a clinically important change in strength for lower extremities, 2 kg to indicate a clinically important change in strength for upper extremities. For weight and body composition outcomes, we considered 3 kg to indicate a clinically important change in body weight (which equals approximately 5% of the average baseline body weight of participants), 5 cm to indicate a clinically important change in girth (arm and thigh), 5 kg/cm^2^ to indicate a clinically important change in body mass index, 5 kg to indicate a clinically important change in fat mass, and 5 cm^2^ to indicate a clinically important change in leg muscle area. For psychological outcomes, we considered 10 points to indicate a clinically important change in the sub scales of the SF-36 quality of life questionnaire [12, 22]. We based these *a priori* estimates on a combination of clinical experience and interpretations in the individual included studies” [12].

“We considered a *p* value of less than 0.1 as statistical significance for heterogeneity between studies [23] and I^2^ < 40 as low heterogeneity, I^2^ > 40–75% moderate, and I^2^ > 75% substantial heterogeneity [19]. In instances of lack of statistical significance for an overall effect, confidence intervals were assessed for potential trends that may suggest movement towards an increase or decrease in overall effect. In instances of statistical significance for heterogeneity, we performed sensitivity analyses and explained potential reasons for heterogeneity” [12].

## Results

**Fig. 1:**
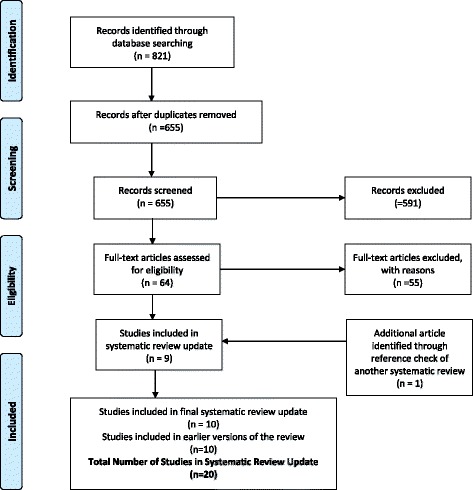
PRISMA Flow Diagram of Included Studies in Progressive Resistive Exercise (PRE) and HIV Systematic Review Update

Ten studies were included in the former systematic review. For this update, we identified a total of 655 citations, 64 of which merited full review of the article. Of the 64 studies reviewed, 10 met the inclusion criteria, one of which was a duplicate publication reporting on the same study [24]. We identified one additional study that met the inclusion criteria after scanning reference lists of pertinent articles, resulting in a total of 10 studies included in this update (Agostini [25], Balasubramanyam [26], Farinatti [27], Fitch [28], Lindegaard [29], Ogalha [30], Perez-Moreno [31], Sakkas [32], Tiozzo [33], Yarasheski [34]) (Fig. 1-PRISMA Flow Diagram). Thus, 20 studies (10 from the previous review and 10 from this update) were included in this systematic review (See Table 1-Selected Characteristics of Included Studies and Additional file 2 for Detailed Characteristics of Included Studies) [25–44]. Eight additional articles were identified as duplicate publications that related to studies included in the review: Kaushik [24] and Fitch [28]; Schroeder [45] Jaque [46] Sattler [47] Schroeder [48] and Sattler [42]; Lox [49] and Lox [40]; Fairfield [50] and Grinspoon [39]; and Driscoll [51] and Driscoll [38]. “In these instances, we extracted outcomes from all available sources but refer to the initial citation or the citation that included our primary outcomes of interest” [12].

**Table 1:** Selected characteristics of included studies in the Progressive Resistive Exercise (PRE) and HIV systematic review (*n* = 20) (for further details, see Additional file 2)

Study	Methods	Sample Size (at baseline)	% Women	% Taking combination ART	Participants (at study completion)	Withdrawal Rate
Agin (2001) [35]	Randomized combined PRE and whey protein vs whey protein alone vs PRE alone [3 groups]	43 (with wasting)	100%	Unknown	30	13/43 (30%)
Agostini (2009) [25]^a, b^	Randomized combined AER + PRE vs diet and aerobic exercise recommendation alone (no exercise) [2 groups]	76	39%	100%	70	6/76 (8%)
Balasubramanyam (2011) [26]^a, b^	Randomized trial with five groups. In this review we compared diet and exercise (lifestyle change) vs usual care (no exercise) [2 groups]	191 (with dyslipidemia)	13%	100%	128 (68 participants in the 2 comparison groups of interest)	63/191 (33%)
Bhasin (2000) [36]	Randomized PRE vs PRE + testosterone vs testosterone only vs no exercise [4 groups]	61 (with involuntary weight loss and low testosterone)	0%	100% taking ARVs (unclear whether it was cART)	49	12/61 (20%)
Dolan (2006) [37]^b^	Randomized constant ARE +PRE vs no exercise [2 groups]	40 (with self-reported and physical evidence of changes in fat distribution)	100%	82% taking ARVs (unclear whether it was cART)	38	2/40 (5%)
Driscoll (2004a) [38]^b^	Randomized combined AER + PRE and metformin vs metformin alone [2 groups]	37 (evidence of fat redistribution and hyperinsulinemia)	20%	100%	25	12/37 (32%)
Farinatti (2010) [27]^a, b^	Randomized constant AER + PRE exercise vs no exercise [2 groups]	27	Not reported	100%	27	0/27 (0%)
Fitch (2012) [28]^a, b^	Randomized constant AER + PRE exercise (LSM) vs AER + PRE exercise + metformin vs no LSM and metformin alone vs versus no exercise (no LSM or metformin) [4 groups]	50 (with metabolic syndrome)	24%	100%	36	14/50 (28%)
Grinspoon (2000) [39]^b^	Randomized PRE + AER vs PRE + AER and testosterone vs testosterone alone vs no exercise [4 groups]	54 (with AIDS-related wasting)	0%	72%	43	11/54 (20%) [4/26 (15%) from the 2 groups of interest]
Lindegaard (2008) [29]^a, b^	Randomized AER vs PRE [2 groups]	20 (with dyslipidemia, lipodystrophy)	0%	100%	18	2/20 (10%)
Lox (1995) [40]^b^	Randomized constant AER vs PRE vs no exercise [3 groups]^c^	22 (aerobic and control groups only)	0%	100% (taking some form of ARV therapy that may or may not have been in combination)	21	1/22 (4%)
Ogalha (2011) [30]^a, b^	Randomized AER+ PRE + nutrition counseling vs nutrition counseling alone [2 groups]	70 (lipodystrophy in 54% of participants)	46%	100%	63	7/70 (10%)
Perez-Moreno (2007) [31]^a, b^	Randomized constant AER+ PRE vs no exercise [2 groups]	27 (prison inmates living with Hepatitis C co-infection)	0%	10%	19	8/27 (30%)
Rigbsy (1992) [41]	Randomized constant AER+ PRE vs no exercise (counselling) [2 groups]	45 (37 HIV+)	0%	Not reported	31 (24 HIV+)	13/37 (35%)
Sakkas (2009) [32]^a^	Randomized PRE+ creatine vs PRE alone [2 groups]	40	0%	75%	33	7/40 (18%)
Sattler (1999) [42]	Randomized PRE+ testosterone vs testosterone only [2 groups]	33	0%	80%	30	3/33 (9%)
Shevitz (2005) [43]	Randomized combined PRE+ nutrition + oxandrolone vs nutrition + oxandrolone vs nutrition alone [3 groups]	50 (with wasting)	30%	80%	47	3/50 (6%)
Spence (1990) [44]	Randomized PRE vs no exercise (control) [2 groups]	24	0%	100% taking AZT	NR	Unknown
Tiozzo (2011) [33]^a, b^	Randomized constant AER + PRE vs no exercise (control) [2 groups]	37	39%	100%	23	14/37 (38%)
Yarasheski (2011) [34]^a, b^	Randomized constant AER+ PRE+ pioglitazone vs pioglitazone only [2 groups]	44 (with insulin resistance, impaired glucose intolerance and central adiposity)	13%	100%	39	5/44 (11%)

^a^Study included in this update of the systematic review;

^b^Study also included in systematic review examining effect of aerobic exercise with adults living with HIV [12] https://bmcinfectdis.biomedcentral.com/articles/10.1186/s12879-016-1478-2; ^c^For this review, PRE and control groups were included in meta-analyses; *PRE* progressive resistive exercise, *AER* aerobic exercise, *NR* not reported, *ART* antiretroviral therapy, *cART* combination antiretroviral therapy, *HAART* highly active antiretroviral therapy, *1RM* 1 repetition maximum, *HR* heart rate, *reps* repetitions, *LSM* lifestyle modification

### Included studies

All 20 included studies were randomized controlled trials. Ten studies included a non-exercising control group [27, 28, 31, 33, 36, 37, 39, 41, 44, 49] and one study included a non-exercising counselling group versus exercise [41]. Twelve studies included groups involving a co-intervention, comparing exercise with diet or nutritional counselling versus diet or nutritional counselling alone (Shevitz [43] diet and oxandrolone; Ogalha [30] nutritional counselling; Balasubramanyam [26] low lipid diet with exercise recommendation; Agostini [25] standard diet); exercise with metformin versus metformin alone (Driscoll [38]; Fitch [28]); exercise with testosterone versus testosterone alone (Grinspoon [39]; Sattler [42]; Bhasin [36]); exercise with whey protein versus whey protein alone (Agin [35]); exercise with creatine versus creatine alone (Sakkas [32]) and exercise with pioglitazone versus pioglitazone alone (Yarasheski [34]).

Six studies included a comparison group of PRE alone, three of which compared PRE versus non-exercising control (Spence [44]; Lox [40]; Bhasin [36]), three compared PRE versus PRE in combination with a co-intervention including whey protein (Agin [35]), testosterone (Bhasin [36]) and creatine (Sakkas [32]), and two compared PRE versus aerobic exercise (Lindegaard [29], Lox [40]).

In 17 studies exercise was supervised and in the remaining three studies, the level of supervision was not reported [26–31, 33–35, 37–39, 41–44, 49]. One study involved a supervised home-based exercise intervention [37]. Nine studies included exercise at supervised facilities such as a hospital [34, 35, 38], exercise laboratory at a university, exercise facility, fitness or wellness centre [29, 30, 33, 42], gymnasium [26], or prison [31]; whereas the location of exercise was not specified in the remaining 11 studies.

### Characteristics of participants

A total of 983 participants were included in the review (number of participants in included studies at baseline). Participants were adults living with HIV with CD4 counts ranging from <100 cells/mm^3^ to >1000 cells/mm^3^. Women comprised approximately 23% of the total number of participants at study completion. The mean age of the participants ranged from 32 to 49 years (inclusion criteria ranged from 18 to 65 years of age).

Three studies (15%) were published before 1996, the timing of the introduction of combination antiretroviral therapy [40, 41, 44] followed by four (20%) between 1998 and 2002 [35, 36, 39, 42], five (17%) between 2004 and 2008 [29, 31, 37, 38, 43] and eight (40%) between 2009 and 2013 [25–28, 30, 32–34]. The majority of participants in 14 studies were taking combination antiretroviral therapy including 72% [39]; 82% [37], 80% [42, 43] and 100% [25–30, 32–34, 38] (Table 1; Additional file 2).

Twelve studies included participants living with concurrent health conditions in addition to HIV. Seven studies included participants with dyslipidemia [26, 29], lipodystrophy [29, 30], changes in fat distribution [37, 38], hyperinsulinemia [38], insulin resistance, glucose intolerance and central adiposity [34], and metabolic syndrome [28]. Four studies included participants with AIDS wasting or involuntary weight loss [35, 36, 39, 43]. One study included prison inmates with Hepatitis C co-infection who were on methadone maintenance [31]. See Table 1; Additional file 2 for more detail on personal characteristics of participants within included studies.

### Outcomes of included studies

All but four included studies (20%) assessed immunological or virological outcomes or both, with CD4 count or viral load [25, 32, 35, 44]. Thirteen of the 20 included studies (65%) assessed cardiorespiratory outcomes [26–31, 33, 37, 38, 41, 43, 44, 49]. Sixteen studies (80%) assessed strength outcomes [27–29, 31–33, 35–39, 41–44, 49]. Nineteen studies (95%) assessed weight and body composition outcomes [25–39, 42–44, 49]. Seven studies (35%) assessed psychological outcomes in the form of mood and life satisfaction, and health-related quality of life. [30, 31, 33, 35, 36, 40, 43]. Safety (assessed by monitoring adverse events) was reported in 13 studies (65%) [26–28, 31, 34–39, 41–43] (see Table 2 for an overview of outcomes assessed in individual studies; see Additional file 3 for a detailed overview of results of outcomes assessed of included studies).

**Table 2:** Outcomes assessed in individual studies included in the Progressive Resistive Exercise (PRE) and HIV systematic review *(*for details of outcomes and authors’ conclusions, see Additional file 3)

Study	Immunological and Virological	Cardiorespiratory	Strength	Weight and Body Composition	Psychological	Adverse Events
Agin (2001) [35]	Not assessed	Not assessed	Upper and lower extremity strength	Weight Body Composition	Health-related quality of life	Assessed
Agostini (2009) [25]^a, b^	Not assessed	Not assessed	Not assessed	Weight Body Composition	Not assessed	Not reported
Balasubramanyam (2011) [26]^a, b^	CD4 count Viral load	VCO2, VO2, respiratory quotient, resting energy expenditure	Not assessed	Weight Body composition	Not assessed	Assessed
Bhasin (2000) [36]	CD4 count Viral load	Not assessed	Upper and lower extremity strength	Weight Body Composition	Health-related quality of life	Assessed
Dolan (2006) [37]^b^	CD4 count Viral load	6MWT VO2max		Weight Body Composition	Not assessed	Assessed
Driscoll (2004a) [38]^b^	CD4 count Viral load	Exercise Time	Upper and lower extremity strength	Weight Body Composition	Not assessed	Assessed
Farinatti (2010) [27]^a, b^	CD4 count	Slope and intercept for HR-workload	Upper and lower extremity strength	Body Composition	Not assessed	Assessed
Fitch (2012) [28]^a, b^	CD4 count Viral load	VO2max and Endurance Time	Upper and lower extremity strength	Body Composition	Not assessed	Assessed
Grinspoon (2000) [39]^b^	CD4 count Viral load	Not assessed	Upper and lower extremity strength	Weight Body Composition	Not assessed	Assessed
Lindegaard (2008) [29]^a, b^	Not reported	VO2max	Upper and lower extremity strength	Weight Body Composition	Not assessed	Not reported
Lox (1995) [40]^b^	CD4 count Viral load	VO2max Heart Rate	Upper and lower extremity strength	Weight Body Composition	Mood and Life Satisfaction	Not reported
Ogalha (2011) [30]^a, b^	CD4 count	VO2max	Not assessed	Weight Body Composition	Quality of Life	Not reported
Perez-Moreno (2007) [31]^a, b^	CD4 count	Workrate maximum HRmax	Upper and lower extremity strength	Body Composition	Quality of Life	Assessed
Rigsby (1992) [41]	CD4 count	Aerobic Capacity Heart Rate Total Time to Voluntary Exhaustion	Upper and lower extremity strength	Not assessed	Not assessed	Assessed
Sakkas (2009) [32]^a^	Not assessed	Fatigue	Upper and Lower Body Strength	Weight Body Composition	Not assessed	Not reported
Sattler (1999) [42]	CD4 count	Not assessed	Upper and Lower Extremity Strength	Weight Body Composition	Not assessed	Assessed
Shevitz (2005) [43]	CD4 count Viral load	Endurance Tolerance	Upper and Lower Extremity Strength	Weight Body Composition	Quality of Life Adjusted Years	Assessed
Spence (1990) [44]	Not assessed	Not assessed	Upper and Lower Extremity Strength	Weight Body Composition	Not assessed	Not reported
Tiozzo (2011) [33]^a, b^	CD4 count Viral Load	VO2max HRmax	Upper and lower extremity strength	Weight Body Composition	Quality of Life	Not reported
Yarasheski (2011) [34]^a, b^	CD4 count Viral Load	Not assessed	Not assessed	Weight Body Composition	Not assessed	Assessed

^a^Study included in this update of the systematic review

^b^Study included in systematic review examining effect of aerobic exercise with adults living with HIV [12] https://bmcinfectdis.biomedcentral.com/articles/10.1186/s12879-016-1478-2

*HRQL* health-related quality of life, *MOS-HIV* Medical Outcomes Study HIV Scale, *VO2max* maximum oxygen consumption, *VCO2* rate of elimination of carbon dioxide, *HRmax* heart rate maximum, *6MWT* 6 min walk test

### Correspondence with authors

Three of five authors we wrote to asking for additional data and clarification responded. “Yarasheski provided additional data including mean change and standard deviations of body mass index outcomes and viral load outcomes [34]. Agostini clarified the intervention included a combination of aerobic and resistive exercise and provided more details on the intervention. Authors indicated they were not able to provide raw data on body weight, fat mass, muscle mass, or waist circumference (data were reported as % increase or decrease) [25]. We requested SF-36 Physical Component Scores (PCS) and Mental Component Scores (MCS) from Tiozzo who responded with data on the eight SF-36 sub-scale scores” [12, 33].

### Risk of bias

**Fig. 2:**
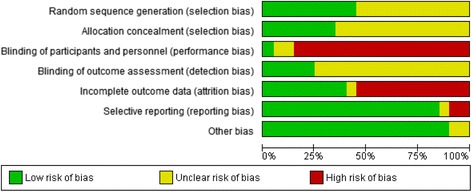
Cochrane Risk of Bias Assessment of Included Studies in Progressive Resistive Exercise (PRE) and HIV Systematic Review Update (*n* = 20 studies)

See Fig. 2 for the risk of bias within included studies. Further detail is provided below.

#### Allocation (Selection Bias)

##### 3.5.1.1.Random sequence generation

Authors from all 20 included studies reported using randomization to allocate participants to the comparison groups. However, an overall *unclear risk for selection bias* exists because authors from 11 of the 20 studies (55%) did not describe the process for randomization [25, 27, 29–32, 34, 41, 43, 44, 49]. Low risk for selection bias was apparent in the remaining nine studies (45%) that described the process for randomization [26, 28, 33, 35–39, 42] (Fig. 2).

##### 3.5.1.2.Allocation concealment

Overall an *unclear risk of selection bias* exists as 13 of the 20 included studies (65%) did not describe the allocation sequence of participants [25–30, 33, 34, 36, 41, 43, 44, 49]. Seven studies (35%) had low risk for selection bias because authors described methods they used to conceal the allocation sequence of participants [31, 32, 35, 37–39, 42] (Fig. 2).

#### Blinding

##### 3.5.2.1.Performance bias

Overall a *high risk of performance bias* exists across the included studies. Seventeen of the 20 included studies (85%) had a high risk for performance bias due to lack of participant blinding to the exercise intervention. Five studies reported single-blinding of outcome assessors to the group allocation [26–28, 31, 32]. In six studies, participants were blinded to co-interventions including metformin [28], creatine [32], oxandrolone [43] and testosterone [36, 39, 42]. However, all of the above studies were considered as high risk for bias because of the inability to blind participants from the exercise intervention (Fig. 2). Unclear or low risk of performance bias was evident in three studies that compared resistive versus aerobic exercise, where both comparison groups involved some type of exercise or when blinding was unclear [25, 29, 32] (Fig. 2).

##### 3.5.2.2.Detection bias

Overall an *unclear risk of detection bias* exists as 12 of the 20 included studies (60%) did not provide enough information about whether the study personnel were blinded to the outcomes assessed. Five studies (25%) had low risk for detection bias because authors reported that outcome assessors were blinded to group allocation [26–28, 31, 32] (Fig. 2).

#### Incomplete outcome data (Attrition Bias)

A total of 195 participants (20%) withdrew from the included studies (195/959 participants at baseline). Withdrawal rates among individual studies ranged from 0% [27, 40] to 38% [33] (Table 1; Additional file 2). Overall a *high risk of attrition bias* exists as 11 of the 20 included studies (55%) reported rates of withdrawal greater than 15%. The remaining nine studies (45%) had low risk of attrition bias with withdrawal rates <15% [25, 27, 29, 30, 34, 37, 42, 43, 49] (Fig. 2).

The rates of withdrawal were similar between comparison groups in most studies. Authors of one study reported that older participants with less familial history of diabetes remained in the study [26]. Almost all of the included studies (19/20; 95%) mentioned participants who were non-adherent with the exercise intervention or withdrew from the study. Spence [44] did not report on any withdrawal of participants. See Table 1 for the proportion of participants who withdrew from individual studies.

Authors reported on adherence to the exercise intervention in seven of the 20 included studies (38%). Adherence rates ranged from 71% to 99% [27, 29, 31–34, 37]. Sakkas [32] reported a 95% adherence rate to the intervention, followed by Yarasheski [34] at 92% Farinatti [27] 87%, and Perez-Moreno [31] 71%. Dolan [37] reported that participants allocated to the exercise group finished 96% of the exercise sessions. Tiozzo [33] reported that participants allocated to the exercise group attended on average 81% of the supervised exercise sessions. Lindegaard [29] reported that adherence to exercise was 96% and 99% in the PRE and aerobic exercise groups, respectively. The six studies that reported on adherence also included exercise interventions that were supervised [27, 29, 31, 33, 34, 39].

#### Selective reporting (Reporting Bias)

Overall a *low risk of reporting bias* exists as most (85%) included studies (17/20) were free of selective outcome reporting because authors provided data on all pre-specified outcomes. Three studies (15%) had incomplete or inconsistent data [25, 26, 30]. Agostini [25] reported outcomes in the form of % increase or decrease in body weight, fat mass, muscle mass and waist circumference. Authors were not able to provide the raw data. Balasubramanyam [26] did not include a complete description of body composition and cardiorespiratory outcomes. In Ogalha [30], data appeared inconsistent across the study tables and authors did not completely report on all outcomes such as viral load (Fig. 2).

#### Other potential sources of bias

Overall a *low risk for other potential sources of bias* exists as most studies (90%) did not appear to have additional issues that could place a study at further risk of bias. Two studies appeared to have unclear risk for additional bias [26, 27]. Balasubramanyam [26] reported receiving funding from the pharmaceutical industry. In Farinatti [27] more participants were allocated to exercise to ensure adequate sample size at study completion in the event that adherence to exercise was low; but it is not clear if this may have skewed findings.

### Meta-analyses - effects of interventions

We conducted 44 meta-analyses across eight sub-group comparisons (10 of which included similar studies) that resulted in 34 unique meta-analyses to this systematic review. We performed meta-analyses for immunological and virological outcomes (CD4 count, viral load), cardiorespiratory outcomes (VO2max, HRmax, exercise time), strength outcomes (chest press, leg press, knee extension, knee flexion), and weight and body composition outcomes (body weight, body mass index, lean body mass, fat mass, leg muscle area, mean arm and thigh girth, waist circumference).

Of the 34 unique meta-analyses, 17 were new to this systematic review update, 10 were updated with additional studies, and seven remained the same as in the former review. Subgroup comparisons of the meta-analyses included 1) PRE or combined PRE and aerobic exercise versus no exercise; 2) PRE versus no exercise; 3) combined PRE and aerobic exercise versus no exercise; 4) PRE (or combined PRE and aerobic exercise) and diet and/or nutrition versus diet and/or nutrition alone; and 5) combined PRE plus testosterone or combined PRE and aerobic exercise plus testosterone versus testosterone alone.

Ten out of the 20 included studies compared PRE or combined PRE and aerobic exercise with a non-exercising control group [27, 28, 31, 33, 36, 37, 39, 41, 44, 49]. Three studies compared PRE with no exercise [36, 44, 49]. Seven studies compared combined PRE and aerobic exercise with no exercise [27, 28, 31, 33, 37, 39, 41]. Two studies compared aerobic with PRE [29, 40] (Table 1; Additional file 2). Driscoll [38] and Fitch [28] included PRE and aerobic exercise combined with metformin compared with metformin alone but we were unable to combine outcomes in meta-analysis. Ogalha [30], Balasubramanyam [26], Shevitz [43], and Agostini [25] included PRE or combined PRE and aerobic exercise combined with a lipid diet and/or nutrition counselling versus diet and/or nutritional counselling alone. Sattler [42], Bhasin [36] and Grinspoon [39] included PRE (or combined PRE and aerobic exercise) combined with testosterone versus testosterone alone. Yarasheski [34] included PRE and aerobic exercise combined with pioglitazone versus pioglitazone alone, Agin [35] included PRE combined with whey protein versus whey protein alone, and Sakkas [32] included PRE combined with creatine versus creatine alone (Table 1; Additional file 2).

Meta-analyses were limited due to the diversity in types of exercise interventions (PRE versus combined PRE and aerobic exercise), level of exercise supervision, outcomes reported, and methodological quality. PRE interventions in the trials varied according to intensity and type of resistive exercise, and combined PRE and aerobic exercise compared to PRE alone. See Table 1 for selected characteristics of included studies and Additional file 2 for a detailed overview of the characteristics of included studies.

### Heterogeneity

Heterogeneity (*p* < 0.1) was present in 16 of the 34 unique meta-analyses (47%). “Reasons for heterogeneity may include differences in the types of participants in relation to antiretroviral use, body composition, comorbidity, gender, type and location of intervention, as well as methods of outcome measurement” [12]. We conducted sensitivity analyses on 11 of the 16 meta-analyses with heterogeneity (see below for specific sensitivity results and reasons for heterogeneity).

### GRADE Ratings and summary of findings table

In the results below, we report on the GRADE rating for seven key outcomes we prioritized as clinically relevant and important to adults living with HIV: viral load; VO2max; upper and lower body strength, body weight; body mass index and fat mass.

#### Immunological and virological outcomes

Sixteen of the 20 included studies (80%) assessed immunological or virological outcomes, or both, in the form of CD4 count or viral load. Nine of the studies included a non-exercising control group, seven of which included interventions with combined PRE and aerobic exercise [27, 28, 31, 33, 37, 39, 41]. Seven studies measured immunological and virological outcomes without a non-exercising control group [26, 28, 30, 34, 38, 42, 43].

##### CD4 count (cells/mm^3^)

Four meta-analyses were performed for CD4 count. Two demonstrated no statistically significant changes in CD4 count between comparison groups, one demonstrated a significant increase in CD4 count favouring exercise and another demonstrated a significant decrease in CD4 count favouring testosterone alone (Table 3). Point estimates were >50 cells/mm^3^ for two meta-analyses comparing exercise to control, which suggested a positive trend towards a potential clinically important improvement in CD4 count with exercise compared with no exercise.

**Table 3:** Results of meta-analyses in Progressive Resistive (PRE) exercise and HIV systematic review: immunological and virological outcomes

Outcomes	Sub-Group Comparison of Meta-Analysis	# of Individual Studies Included in Meta-Analysis	Number of Participants Included in Meta-Analysis	Weighted Mean Difference (WMD)	95% Confidence Interval	*P* value of overall effect	I^2^ statistic (*p* value for heterogeneity)	Interpretation
CD4 count (cells/mm^3^)	PRE or combined PRE and aerobic exercise compared with no exercise	8 studies (Dolan 2006 [37]; Farinatti 2010 [27]; Fitch 2012 [28]; Grinspoon 2000 [39]; Lox 1995 [40]; Perez-Moreno 2007 [31]; Rigsby 1992 [41]; Tiozzo 2011 [33])	195	63.95 cells/mm^3,b^	12.42, 115.48	0.01^a^	70% (*p* = 0.001)	Significant increase in CD4 count among exercisers compared with non-exercisers. “Confidence interval indicates a positive trend towards an improvement in CD4 count among exercisers.” [12].
	Combined PRE and aerobic exercise compared with no exercise	7 studies (Dolan 2006 [37]; Farinatti 2010 [27]; Fitch 2012 [28]; Grinspoon 2000 [39]; Perez-Moreno 2007 [31]; Rigsby 1992 [41]; Tiozzo 2011 [33])	173	57.82 cells/mm^3^	−1.27, 116.91	0.06	74% (*p* < 0.0001)	“No difference in change in CD4 count among exercisers compared with non-exercisers. Confidence interval indicates a positive trend towards an improvement in CD4 count among exercisers.” [12].
	PRE (or combined PRE and aerobic exercise) and diet and/or nutrition counselling group compared with diet and/or nutrition counselling alone.	3 studies (Balasubramanyam 2011 [26]; Ogalha 2011 [30]; Shevitz 2005 [43])	162	20.18 cells/mm^3^	−21.49, 61.85	0.34	78% (*p* = 0.01)	No difference in change in CD4 count among exercisers compared with non-exercisers.
	PRE (or combined PRE and aerobic exercise) and testosterone compared with testosterone alone	2 studies (Grinspoon 2000 [39]; Sattler 1999 [42])	51	−32.13 cells/mm^3^	−56.96, −7.30	0.01^a^	0% (*p* = 0.96)	Significant decrease in CD4 count among exercisers taking testosterone compared with those taking testosterone only.
Viral Load (log10 copies)	Combined PRE and aerobic exercise group compared with compared with no exercise	4 studies (Dolan 2006 [37]; Fitch 2012 [28]; Grinspoon 2000 [39]; Tiozzo 2011 [33])	99	0.12 log10 copies	−0.23, 0.46	0.51	0% (*p* = 0.46)	“No difference in change in viral load among exercisers compared with non-exercisers.” [12].
	PRE (or combined PRE and aerobic exercise) plus diet and/or nutrition compared with diet and/or nutrition only	2 studies (Balasubramanyam 2011 [26]; Shevitz 2005 [43])	99	0.37 log10 copies	−1.43, 2.17	0.40	31% (*p* = 0.23)	No difference in change in viral load among exercisers compared with non-exercisers.

^a^Indicates statistical significance; ^b^indicates potential clinically important improvement

*Heterogeneity*: Three of the four meta-analyses were statistically significant for heterogeneity (*p* < 0.1). Sensitivity analyses were conducted for the three meta-analyses. While removing Farinatti [27] and Shevitz [43] reduced heterogeneity; sensitivity analyses did not change the overall effect of exercise on CD4 count beyond clinical importance.

##### 3.8.1.2.Viral load (log10copies)

Three meta-analyses were performed for viral load, one of which included the same studies. Meta-analyses demonstrated no difference in change in viral load for participants in the combined PRE and aerobic exercise intervention group compared with the non-exercising control group as well as the combined PRE and aerobic exercise group with diet and/or nutrition compared with the non-exercising diet and/or nutrition only group (Table 3). None of the meta-analyses were significant for heterogeneity.

GRADE rating: We are moderately confident in the non-significant effect estimate of 0.12 log10copies demonstrating no difference in change in viral load comparing PRE exercise (or combined PRE and aerobic exercise). “The true effect is likely to be close to the estimate of the effect, but there is a possibility that it may be substantially different. This outcome was downgraded from high to moderate GRADE quality of evidence due to incomplete outcome data (withdrawals of included studies were >15%)” [12] (see Additional file 4 – GRADE Summary of Findings Table).

#### Cardiorespiratory outcomes

Thirteen of the 20 included studies (65%) assessed cardiorespiratory outcomes, seven of which compared PRE or combined PRE and aerobic exercise to no exercise [27, 28, 31, 33, 37, 41, 49] and two of which compared PRE to aerobic exercise [29, 49]. Driscoll [38], Fitch [28], and Sakkas (2009) measured cardiorespiratory outcomes but compared exercise to metformin, or creatine [32]. Shevitz [43], Ogalha [30], and Balasubramanyam [26] measured cardiorespiratory outcomes but compared exercise combined with diet and/or nutritional counselling (Table 1; Additional file 2).

##### VO2max

Two meta-analyses were performed for VO2max, one of which included the same studies. Results showed a significant and potential clinically important improvement in change of VO2max of 3.71 mL/kg/min for participants in the aerobic exercise intervention group compared with the non-exercising control group (Table 4).

**Table 4:** Results of meta-analyses in Progressive Resistive Exercise (PRE) and HIV systematic review: cardiorespiratory outcomes

Outcomes	Sub-Group Comparison of Meta-Analysis	# of Individual Studies Included in Meta-Analysis	Number of Participants Included in Meta-Analysis	Weighted Mean Difference (WMD)	95% Confidence Interval	*P* value of overall effect	I^2^ statistic (*p* value for heterogeneity)	Interpretation
VO2max (ml/kg/min)	PRE or combined PRE and aerobic exercise compared with no exercise	3 studies (Dolan 2006 [37]; Fitch 2012 [28]; Tiozzo 2011 [33])	82	3.71 ml/kg/min^b^	1.73, 5.70	0.0002^a^	0% (*p* = 0.84)	“Significant (and potential clinically important) improvement in change in VO2max among exercisers compared with non-exercisers.” [12].
Maximum Heart Rate (bpm)	PRE or combined PRE and aerobic exercise compared with no exercise	3 studies (Lox 1995 [40]; Perez-Moreno 2007 [31]; Rigsby 1992 [41])	65	−5.23 beats per minute	−23.84, 13.37	0.58	97% (*p* < 0.00001)	“No significant difference in change in heart rate maximum among exercisers compared with non-exercisers.” [12].
	Combined PRE and aerobic exercise group compared with no exercise	2 studies (Perez-Moreno 2007 [31]; Rigsby 1992 [41])	43	−4.91 beats per minute	−34.13, 24.30	0.74	99% (*p* < 0.00001)	“No significant difference in change in heart rate maximum among exercisers compared with non-exercisers.” [12].
Exercise Time (min)	Combined aerobic and PRE group compared with no exercise	3 studies (Dolan 2006 [37]; Fitch 2012 [28]; Rigbsy 1992 [41])	83	3.29 min	0.10, 6.49	0.04^a^	97% (*p* < 0.00001)	“Significant increase in exercise time among exercisers compared with non-exercisers.” [12].

^a^Indicates statistical significance; ^b^indicates potential clinically important improvement; bpm = beats per minute

*Heterogeneity*: There was no statistical significance for heterogeneity.

GRADE rating: We are moderately confident in the effect estimate demonstrating a significant increase of 3.71 ml/kg/min for VO2max comparing PRE exercise (or combined PRE and aerobic exercise). The true effect is likely to be close to the estimate of the effect but there is a possibility that it may be substantially different. This outcome was downgraded from high to moderate GRADE quality of evidence because the lower level of the confidence interval did not cross the estimated clinically important change in VO2max (despite the estimate surpassing our hypothesized clinically important change in VO2max of 2 ml/kg/min) (see Additional file 4 – GRADE Summary of Findings Table).

##### 3.8.2.2.Maximum heart rate (HRmax)

Two meta-analyses were performed and showed no significant difference in change in HRmax for participants in the PRE or combined PRE and aerobic exercise group compared with the non-exercising control; and combined PRE and aerobic exercise group compared with non-exercising control (Table 4).

*Heterogeneity*: Heterogeneity was present in both meta-analyses (*p* < 0.1). Sensitivity analyses were conducted for the meta-analysis that had greater than two studies. Removing any of the three included studies did not reduce heterogeneity. “Reasons for heterogeneity may be due to differences in characteristics of participants in the included studies; participants in Perez-Moreno [31] were all in prison and co-infected with Hepatitis C.” [12].

##### 3.8.2.3.Exercise time

Two meta-analyses were performed, one of which included the same studies. Similar to our aerobic review, “meta-analysis demonstrated a significant increase in exercise time of 3.29 min for participants in the combined PRE and aerobic exercise group compared with the non-exercising control group (Table 4); the point estimate did not reach the 5 min threshold for clinical importance” [12].

*Heterogeneity*: Meta-analyses were statistically significant for heterogeneity (*p* < 0.1). Removing Rigsby [41] removed heterogeneity and the overall effect remained significant, but was reduced to 1.72 min [95% CI: 1.03, 2.42] among exercisers compared with control (not shown). Reasons for heterogeneity may be due to differences in characteristics of participants in the included studies.

See Table 2 for outcomes assessed and Additional file 3 for results for outcomes assessed in individual studies unable to be combined in meta-analyses.

#### Strength outcomes

Sixteen of the 20 included studies (80%) assessed strength outcomes [27–29, 31–33, 35–39, 41–44, 49]. We performed 10 meta-analyses, four of which included duplicate studies. Results demonstrated significant improvements in upper and lower body strength as determined by increases in 1-repetition maximum for chest press, and knee flexion; and a non-significant trend towards improvement (increases) in 1-RM for leg press and knee extension for participants in the combined PRE and aerobic group versus non-exercising control group (Table 5). We conducted two meta-analyses comparing combined exercise and testosterone compared with testosterone alone. Results indicated a non-significant trend towards greater increases in strength among participants in the combined exercise and testosterone group compared with participants in the testosterone alone group for knee flexion and extension (Table 5).

**Table 5:** Results of meta-analyses in Progressive Resistive Exercise (PRE) and HIV systematic review: strength outcomes

Outcomes	Sub-Group Comparison of Meta-Analysis	# of Individual Studies Included in Meta-Analysis	Number of Participants Included in Meta-Analysis	Weighted Mean Difference (WMD)	95% Confidence Interval	*P* value of overall effect	I^2^ statistic (*p* value for heterogeneity)	Interpretation
Chest Press (1-RM)	Combined PRE and aerobic exercise group compared with no exercise	2 studies (Fitch 2012 [28]; Tiozzo 2011 [33])	44	11.86 kg 1-RM^b^	2.37, 21.36	0.01^a^	46% (*p* = 0.18)	“Significant and potential clinically important improvement in change in chest press 1-repetition maximum among exercisers compared with non-exercisers.” [12].
Knee Flexion (1-RM)	Combined PRE and aerobic exercise group compared with no exercise	3 studies (Dolan 2006 [37], Fitch 2012 [28]; Grinspoon 2000) [39]	81	10.46 kg 1-RM^b^	1.64, 19.29	0.02^a^	91% (*p* < 0.00001)	“Significant and potential clinical important improvement in change in knee flexion 1-repetition maximum among exercisers compared with non-exercisers” [12].
	PRE (or combined PRE and aerobic exercise) and testosterone compared with testosterone alone	2 studies (Grinspoon 2000 [39]; Sattler 1999 [42])	51	4.67 kg 1-RM	−1.98, 11.31	0.17	89% (*p* = 0.002)	Non-significant trend towards a greater increase in knee extension 1-RM among exercisers taking testosterone compared with non-exercisers taking testosterone only.
Leg Press (1-RM)	Combined PRE and aerobic exercise group compared with no exercise	2 studies (Fitch 2012 [28]; Tiozzo 2011 [33])	44	50.96 kg 1-RM^b^	−13.01, 114.92	0.12	88% (*p* = 0.004)	“Non-significant trend towards an increase in leg press 1-RM among exercisers compared with non-exercisers.” [12].
Knee Extension (1-RM)	Combined PRE and aerobic exercise group compared with no exercise	3 studies (Dolan 2006 [37]; Fitch 2012 [28]; Grinspoon 2000 [39])	81	20.58 kg 1-RM^b^	−4.69, 45.86	0.11	95% (*p* < 0.00001)	“Non-significant trend towards an increase in knee extension 1-RM among exercisers compared with non-exercisers.” [12].
	PRE (or combined PRE and aerobic exercise) and testosterone compared with testosterone alone	2 studies (Grinspoon 2000 [39]; Sattler 1999 [42])	51	13.09 kg 1-RM^b^	−9.94, 36.11	0.27	97% (*p* < 0.00001)	Non-significant trend towards a greater increase in knee extension 1-RM among exercisers taking testosterone compared with non-exercisers taking testosterone alone.

*1-RM* 1 repetition maximum, *PRE* progressive resistive exercise

^a^Indicates statistical significance; ^b^indicates potential clinically important change in outcome

Five of the six point estimates for upper and lower extremity strength were greater than 2 kg and 5 kg respectively indicating a clinically important greater increase with exercise compared with non-exercise.

*Heterogeneity*: Heterogeneity was present in five meta-analyses. “Removing Grinspoon [39] from the combined PRE and aerobic exercise versus control comparison reduced heterogeneity (*p* = 0.95) for knee extension and the overall effect became significant for exercise compared with no exercise (not shown). Reasons for heterogeneity may be attributed to differences in study participants. Participants in Grinspoon [39] had signs of AIDS-related wasting” [12].

GRADE ratings: Similar to our aerobic exercise review, “our confidence is limited in the effect estimate of a significant increase of 11.86 kg for 1-repetition maximum for chest press comparing PRE exercise (or combined PRE and aerobic exercise) with non-exercising control. The true effect may be substantially different from the estimate of effect. This outcome was downgraded from high to low on the GRADE quality of evidence due to incomplete outcome data (withdrawals of included studies were >15%), publication bias suspected, and moderate heterogeneity (I^2^=46%). However, the estimate demonstrated a significant effect for improvement in chest press and the lower limit of the confidence interval surpassed our hypothesized clinically important change in upper body strength” [12] (see Additional file 4 – GRADE Summary of Findings Table).

“We have very little confidence in the effect estimate of a non-significant increase of 50.96 kg for 1-repetition maximum for leg press comparing PRE exercise (or combined PRE and aerobic exercise) with non-exercising control. The true effect is likely to be substantially different from the estimate of effect. This outcome was downgraded from high to very low on the GRADE quality of evidence due to incomplete outcome data (withdrawals of included studies were >15%), publication bias was suspected, and there was substantial heterogeneity (I^2^=88%). Furthermore, the confidence intervals cross the clinically important improvement and deterioration for change in lower body strength” [12] (see Additional file 4 – GRADE Summary of Findings Table).

#### Weight and body composition outcomes

Nineteen out of the 20 included studies (95%) assessed weight and body composition outcomes [25–39, 42–44, 49].

##### 3.8.4.1.Weight

Fifteen studies assessed body weight [26, 29, 30, 32–39, 42–44, 49]. Five meta-analyses were performed. Meta-analyses demonstrated a significant increase in body weight of 2.50 kg for participants in the PRE or combined PRE and aerobic exercise group and a significant and potentially clinically important increase in body weight of 4.24 kg among participants in the PRE group compared with aerobic exercise group. No differences were found for change in mean body weight for participants in the combined aerobic and PRE group compared with the non-exercising control group; the combined PRE (or combined PRE and aerobic exercise) and diet/nutrition counselling group compared with diet/nutritional counselling only group; and the combined PRE and testosterone group compared with the testosterone only group (Table 6).

**Table 6:** Results of meta-analyses in Progressive Resistive Exercise (PRE) and HIV systematic review: weight and body composition outcomes

Outcomes	Sub-Group Comparison of Meta-Analysis	# of Individual Studies Included in Meta-Analysis	Number of Participants Included in Meta-Analysis	Weighted Mean Difference (WMD)	95% Confidence Interval	*P* value of overall effect	I^2^ statistic (*p* value for heterogeneity)	Interpretation
Mean Body Weight (kg)	PRE or combined PRE and aerobic exercise compared with no exercise	5 studies (Dolan 2006 [37]; Grinspoon 2000 [39]; Lox 1995 [40]; Spence 1990 [44]; Tiozzo 2011 [33])	129	2.50 kg	0.32, 4.67	0.02^a^	76% (*p* = 0.002)	Significant increase in body weight among exercisers compared with non-exercisers.
	PRE compared with no exercise	2 studies (Lox 1995 [40]; Spence 1990 [44])	46	4.24 kg^b^	1.82, 6.66	0.0006^a^	39% (*p* = 0.20)	Significant and potential clinically important increase in body weight among exercisers compared with non-exercisers.
	Combined PRE and aerobic exercise compared with no exercise	3 studies (Dolan 2006 [37]; Grinspoon 2000 [39]; Tiozzo 2011 [33])	83	0.81 kg	−0.94, 2.56	0.37	19% (*p* = 0.29)	“No difference in change in body weight among exercisers compared with non-exercisers.” [12].
	PRE (or combined PRE and aerobic exercise) and diet and/or nutrition counselling group compared with diet and/or nutrition counselling alone.	3 studies (Balasumbramanyam 2011 [26]; Ogalha 2011 [30]; Shevitz 2005 [43])	162	−0.67 kg	−4.25, 2.92	0.72	93% (*p* < 0.00001)	“No difference in change in body weight for participants in the combined exercise and diet or nutrition counselling group compared with the diet or nutrition counselling alone group.” [12].
	PRE (or combined PRE and aerobic exercise) and testosterone compared with testosterone alone	2 studies (Grinspoon 2000 [39]; Sattler 1999 [42])	51	0.42 kg	−0.92, 1.77	0.54	0% (*p* = 0.48)	No difference in change in body weight for exercisers taking testosterone compared with those taking testosterone only.
Body Mass Index (kg/m^2^)	PRE or combined PRE and aerobic exercise compared with no exercise	5 studies (Dolan 2006 [37]; Farinatti 2010 [27]; Fitch 2012 [28]; Lox 1995 [40]; Tiozzo 2011 [33])	131	0.40 kg/m^2^	−0.22, 1.03	0.21	34% (*p* = 0.19)	“No difference in change in body mass index among exercisers compared with non-exercisers.” [12].
	Combined PRE and aerobic exercise compared with no exercise	4 studies (Dolan 2006 [37]; Farinatti 2010 [27]; Fitch 2012 [28]; Tiozzo 2011 [33])	109	0.21 kg/m^2^	−0.27, 0.68	0.40	0% (*p* = 0.40)	“No difference in change in body mass index among exercisers compared with non-exercisers.” [12].
	PRE (or combined PRE and aerobic exercise) and diet and/or nutrition counselling group compared with diet and/or nutrition counselling alone	3 studies (Balasubramanyam 2011 [26]; Ogalha 2011 [30]; Shevitz 2005 [43])	162	−0.55 kg/m^2^	−1.22, 0.12	0.11	83% (*p* = 0.002)	No difference in change in body mass index for participants in the combined PRE and diet or nutrition counselling group compared with the diet or nutrition counselling only group.
Lean Body Mass (kg)	PRE or combined PRE and aerobic exercise compared with no exercise	4 studies (Farinatti 2010 [27]; Grinspoon 2000 [39]; Lox 1995 [40]; Perez-Moreno 2007 [31])	90	2.14 kg	−0.11, 4.39	0.06	59% (*p* = 0.06)	“No difference in change in lean body mass among exercisers compared with non-exercisers.” [12].
	Combined PRE and aerobic exercise compared with no exercise	3 studies (Farinatti 2010 [27], Grinspoon 2000 [39]; Perez-Moreno 2007 [31])	68	1.23 kg	−0.62, 3.08	0.19	17% (*p* = 0.30)	“No difference in change in lean body mass among exercisers compared with non-exercisers.” [12].
	PRE (or combined PRE and aerobic exercise) and testosterone compared with testosterone alone	2 studies (Grinspoon 2000 [39]; Sattler 1999 [42])	51	0.64 kg	−0.97, 2.26	0.44	0% (*p* = 0.63)	No difference in change in lean body mass for exercisers taking testosterone compared with those taking testosterone alone.
Leg Muscle Area (cm^2^ or mm^2)^	Combined PRE and aerobic exercise compared with no exercise	2 studies (Dolan 2006 [37]; Grinspoon 2000 [39])	60	4.79 cm^2^	2.04, 7.54	0.0007^a^	11% (*p* = 0.29)	Significant increase in leg muscle area among exercisers compared with non-exercisers.
	PRE (or combined PRE and aerobic exercise) and testosterone compared with testosterone alone	2 studies (Grinspoon 2000 [39]; Sattler 1999 [42])	51	56.09 mm^2^	−359.53, 471.72	0.79	0% (*p* = 0.67)	No difference in change in leg muscle area for exercisers taking testosterone compared with those taking testosterone only.
Fat Mass (kg)	PRE or combined PRE and aerobic exercise compared with no exercise	4 studies (Dolan 2006 [37]; Fitch 2012 [28]; Grinspoon 2000 [39]; Lox 1995 [40])	103	0.36 kg	−0.50, 1.23	0.41	0% (*p* = 0.53)	“No difference in change in fat mass among exercisers compared with non-exercisers.” [12].
	Combined PRE and aerobic exercise compared with no exercise	3 studies (Dolan 2006 [37]; Fitch 2012 [28]; Grinspoon 2000 [39])	81	0.18 kg	−0.74, 1.10	0.70	0% (*p* = 0.63)	“No difference in change in fat mass among exercisers compared with non-exercisers.” [12].
	PRE (or combined PRE and aerobic exercise) and testosterone compared with testosterone alone	2 studies (Grinspoon 2000 [39]; Sattler 1999 [42])	51	−0.73 kg	−1.50, 0.04	0.06	0% (*p* = 0.86)	No difference in change in fat mass for exercisers taking testosterone compared with those taking testosterone only.
Waist Circumference (cm)	Combined PRE and aerobic exercise compared with no exercise	3 studies (Dolan 2006 [37]; Fitch 2012 [28]; Tiozzo 2011 [33])	82	−1.33 cm	−4.21, 1.54	0.36	37% (*p* = 0.21)	“No difference in change in waist circumference among exercisers compared with non-exercisers.” [12].
Arm and Thigh Girth (cm)	PRE compared with no exercise	2 studies (Lox 1995 [40]; Spence 1990 [44])	46	7.91 cm^b^	2.18, 13.65	0.007^a^	67% (*p* = 0.08)	Significant and potential clinically important increase in arm and thigh girth among exercisers compared with non-exercisers.

^a^Indicates statistical significance; ^b^indicates potential clinically important change in outcome

*Heterogeneity*: Heterogeneity was present in two of the five meta-analyses (*p* < 0.1). Removing Dolan [37] from the PRE or combined PRE and aerobic exercise group reduced heterogeneity (*p* = 0.02) and increased the overall estimate of increase in body weight from 2.50 kg to 3.46 kg (not shown). Removing Balasubramanyam [26] from the combined PRE and diet/nutritional counselling versus non-exercise control comparison reduced heterogeneity (*p* = 0.12) but the overall effect remained non-significant (not shown). Reasons for heterogeneity may be due to differences in the comorbidity of participants in the included studies. In Balasubramanyam [26], participants had dyslipidemia, in Ogalha [30], 54% of participants had lipodystrophy and in Dolan [37] participants had self-reported changes in fat distribution.

GRADE rating: We have very little confidence in the effect estimate of a significant increase of 2.5 kg for body weight comparing PRE exercise (or combined PRE and aerobic exercise) with no exercise. “The true effect is likely to be substantially different from the estimate of effect. This outcome was downgraded from high to very low on the GRADE quality of evidence due to incomplete outcome data (withdrawals of included studies were >15%)” [12], and there was substantial heterogeneity (I^2^ = 76%). The estimate surpassed our hypothesized clinically important change in body weight, but the lower level of the confidence interval does not surpass the threshold for clinically important change in weight (see Additional file 4 – GRADE Summary of Findings Table).

##### 3.8.4.2.Body composition

Nineteen out of the 20 included studies (95%) assessed body composition [25–39, 42–44, 49]. Sixteen meta-analyses were performed, each for body mass index, lean body mass, fat mass, arm and thigh girth, leg muscle area, and waist circumference. Three of the 16 meta-analyses were duplicate and included the same studies.

##### 3.8.4.3.Body mass index

Results demonstrated no difference in change in body mass index for three comparisons of participants in the PRE or combined PRE and aerobic exercise group compared with non-exercising control; combined PRE and aerobic exercise group compared with non-exercising control and combined PRE (or combined PRE and aerobic exercise) and diet/nutrition counselling group compared with diet/nutritional counselling group only (Table 6).

GRADE rating: We are moderately confident in the effect estimate of a non-significant increase of 0.40 kg/m^2^ for body mass index comparing PRE (or combined PRE and aerobic exercise) with no exercise. “The true effect is likely to be close to the estimate of effect, but there is a possibility that it is substantially different” [12]. This outcome was downgraded on the GRADE quality of evidence because publication bias was suspected. However, withdrawal rates among the majority of included studies were <15% (see Additional file 4 – GRADE Summary of Findings Table).

##### 3.8.4.4.Lean body mass

Meta-analyses demonstrated no difference in change in lean body mass for three comparisons of participants in the PRE or combined PRE and aerobic exercise group compared with non-exercising control; combined PRE and aerobic exercise group compared with non-exercising control and combined PRE (or combined PRE and aerobic exercise) and testosterone group compared with the testosterone only group (Table 6).

##### 3.8.4.5.Leg muscle area

Similar to our aerobic systematic review, “results demonstrated a significant increase in change in leg muscle area of 4.79 cm^2^ among participants in the combined PRE and aerobic exercise group compared with the non-exercising control group” [12]. No difference was found in leg muscle area for participants in the PRE (or combined PRE and aerobic exercise) and testosterone group compared with the testosterone only group (Table 6).

##### 3.8.4.6.Fat mass

Results demonstrated no difference in change in fat mass for three comparisons of participants in the PRE or combined PRE and aerobic exercise group compared with non-exercising control; combined PRE and aerobic exercise group compared with non-exercising control, and combined PRE (or combined PRE and aerobic exercise) and testosterone group compared with the testosterone alone group (Table 6).

GRADE rating: We are moderately confident with the effect estimate of a non-significant increase of 0.36 kg in fat mass comparing PRE (or combined PRE and aerobic exercise) with non-exercising control. “The true effect is likely to be close to the estimate of effect, but there is a possibility that it is substantially different. This outcome was downgraded on the GRADE quality of evidence due to incomplete outcome data (withdrawals of included studies were >15%)” [12] (see Additional file 4 – GRADE Summary of Findings Table).

##### 3.8.4.7.Waist circumference

No significant differences were found in change in waist circumference for participants in the combined PRE and aerobic exercise group compared with no exercise (Table 6).

##### 3.8.4.8.Arm and thigh girth

Results demonstrated a significant increase in change in arm and thigh girth of 7.91 cm among participants in the PRE group compared with the aerobic exercise group. The point estimate is greater than 5 cm indicating a potential clinically important greater increase in girth among PRE versus aerobic exercisers (Table 6).

*Heterogeneity*: Heterogeneity was present in three meta-analyses for body mass index; lean body mass; and arm and thigh girth (*p* < 0.1). We conducted sensitivity analyses for two meta-analyses that included more than two studies (body mass index and lean body mass). Removing Farinatti [27] or Lox [40] from the PRE or combined PRE and aerobic exercise intervention versus non-exercising control comparison reduced heterogeneity for body mass index (*p* = 0.24 or *p* = 0.32 respectively). Removing Farinatti [27] resulted in a significant increase in lean body mass of 3.10 kg (95% CI: 1.20, 5.00) among exercisers compared with non-exercisers (not shown). Removing Balasubramanyam [26] from the combined PRE (or combined PRE and aerobic exercise) and diet/nutritional counselling versus non-exercise control comparison reduced heterogeneity for body mass index (*p* = 0.43) but the overall effect remained non-significant (not shown). “Reasons for heterogeneity may be due to differences in participants in the included studies” [12]. For example, in Balasubramanyam [26], participants had dyslipidemia.

#### Psychological outcomes

Seven of the 20 included studies (35%) assessed psychological outcomes in the form of mood and life satisfaction, and health-related quality of life [30, 31, 33, 35, 36, 40, 43]. Due to the diversity of outcomes measured we were unable to perform a meta-analysis. Results from five individual studies demonstrated improvements in quality of life, and mood and life satisfaction scores among the exercise groups [30, 31, 33, 35, 40]. Lox [40] reported higher life satisfaction with aerobic versus PRE. Bhasin [36] reported no change in HRQL scores in either the testosterone or combined testosterone and exercise groups. Shevitz [43] reported no significant change in Quality of Life Adjusted Years within groups but reported the increase was greatest with combined PRE and nutrition compared with nutrition alone.

#### Adverse events (Safety)

Authors in 13 of the 20 studies (65%) reported safety in the form of monitoring adverse events [26–28, 31, 34–39, 41–43] (Table 2; Additional file 3). We could not perform a meta-analysis due to the dearth and inconsistency of reporting adverse events. Adverse events were reported in seven of the 20 studies, none of which were attributed to exercise or considered serious [26, 28, 35–37, 41, 42] Six studies reported no serious adverse events, health problems, or complications [27, 31, 34, 38, 39, 43]. Authors from other studies did not report on outcomes of adverse events (Table 2; Additional file 3).

## Discussion

Meta-analyses suggest that performing PRE, or a combination of PRE and aerobic exercise for at least 20 min three times per week for at least six weeks can improve cardiorespiratory fitness (maximum oxygen consumption, exercise time), weight, body composition (leg muscle area, arm and thigh girth), and strength (chest press, knee flexion). Results suggest that PRE is safe for medically stable adults living with HIV. This result is based on few reports of adverse events with exercise within the included studies as well as the lack of change in CD4 count and viral load. “Results are based on participants who completed the exercise interventions and for whom there were adequate follow-up data” [12].

Ten studies were incorporated into the update of this systematic review, seven of which we included in meta-analyses [26–28, 30, 31, 33, 34]. As a result, we were able to perform 17 new meta-analyses for CD4 count, viral load, VO2max, maximum heart rate, strength (chest press, leg press, knee extension and knee flexion), weight, body mass index, lean body mass, leg muscle area, and waist circumference. Also, by incorporating these additional studies we were able to update 10 meta-analyses from our previous review [10].

To our knowledge, this is the first review to conduct sub-group analyses comparing testosterone as a co-intervention with exercise. Testosterone did not appear to significantly enhance outcomes for weight, body composition, or strength, although there was a non-significant trend towards greater improvements in strength among exercisers taking testosterone compared with those taking testosterone alone. Similarly, co-interventions of diet and/or nutrition counseling did not appear to significantly enhance outcomes for weight or body composition beyond exercise alone.

Results of the meta-analyses demonstrated statistically significant improvements in cardiorespiratory fitness (maximum oxygen consumption (VO2max); exercise time), strength (chest press, knee flexion), weight, and body composition (increase in leg muscle area and arm and thigh girth).

Results for cardiorespiratory outcomes showed potential clinically important improvements in VO2max among exercisers versus non-exercisers. While fewer studies assessed cardiorespiratory outcomes in this PRE-focused review (65%) compared with our aerobic exercise systematic review (83%), these findings similarly suggest benefits to cardiorespiratory health. Results for strength outcomes also demonstrated potential clinically important improvements in chest press and knee flexion with combined PRE and aerobic exercise versus no exercise. These findings suggest a combination of PRE and aerobic exercise is most useful for maximizing benefits to cardiovascular health and strength for adults living with HIV [52].

Weight and body composition results reached clinically important increases in body weight, and arm and thigh girth for only PRE compared with non-exercise, suggesting greater increases in weight and girth with resistive exercise. Other statistically significant increases in weight and leg muscle area also were seen with combined PRE and aerobic exercise compared with non-exercise. Meta-analyses for arm and thigh girth and leg muscle area were new to this review. Interpreting changes in weight and body composition in the context of HIV have changed since the widespread use of combination antiretroviral therapy. The findings may be considered as favorable reflecting an increase in strength and muscle mass for adults with HIV. Future reviews may consider sub-group analyses comparing outcomes from studies published prior to versus after the introduction of combination antiretroviral therapy.

We were unable to conduct meta-analyses for psychological outcomes in this review. A recent systematic review focused on aerobic exercise reported significant and clinically important improvements in health-related quality of life and symptoms of depression with exercise versus no exercise [12]. Similarly, results from individual studies in this review demonstrated significant improvements in quality of life and mood and life satisfaction scores.

Overall, exercise appears to be safe for adults with HIV. No significant differences were found for the majority of meta-analyses for CD4 count and viral load outcomes, suggesting PRE has little impact on immune or virological status. Similarly there were minimal adverse effects documented in the included studies and few attributed to PRE. These results are parallel to former versions of this review and our systematic review focused on aerobic exercise [10, 12].

Twelve of the included studies in this review involved combined resistive and aerobic exercise interventions highlighting an increasing trend of combined exercise interventions in the literature [25–28, 30, 31, 33, 34, 37–39, 41]. Results of this PRE systematic review are distinct from earlier systematic reviews focused on aerobic exercise or combined PRE and aerobic exercise [12, 53]. This review includes seven randomized controlled trials comparing pure PRE to non-exercising control or an alternative intervention. Furthermore, results provide new insight into the greater potential clinically important effect of PRE on weight and body composition, and the impact of co-interventions such as testosterone with exercise compared with testosterone alone. In addition, more studies (80%) in this review assessed strength outcomes than the aerobic review (46%) providing a better understanding about improvements in strength with PRE. Collectively, results of this review are complementary to a previous systematic review focused on aerobic exercise using the Cochrane Collaboration protocol, highlighting the benefits of combined aerobic and PRE for adults living with HIV [12].

“Meta-analyses were limited due to variation in outcome measures used, comparison groups and types of interventions, enabling only two or three studies to be included in most meta-analyses [54]. Studies included in this review demonstrated a high risk of performance bias due to the inability to blind participants to the exercise intervention. This subsequently resulted in low GRADE ratings for quality of evidence (Additional file 4). This may have resulted in a Hawthorne effect, whereby participants might perceive greater benefits associated with exercise based on the expectation that exercise should be linked to positive outcomes. Furthermore, lack of assessor blinding may have resulted in assessor bias whereby assessors may have measured outcomes in favor of the exercise intervention. High risk of attrition bias was also evidence due to incomplete data as many studies had withdrawal rates >15%. Individual studies in this review included small sample sizes and high withdrawal or non-adherence rates” (0%–38%) [12]. “Participants who withdrew from the interventions were often excluded from the results, resulting in a per protocol analysis. Nevertheless, authors commonly reported similar withdrawal rates across comparison groups, and characteristics of participants were similar among those who withdrew and remained in the study, minimizing the potential for migration bias” [12].

Similar to our previous review, “the majority of study participants were men between the ages of 18-65 years limiting the ability to generalize results to women and older adults with HIV and multi-morbidity” [12]. Because the longest PRE intervention was 52 weeks, the long-term effect of exercise is unclear [12]. Furthermore, there were no studies in this review that assessed the impact of resistance exercise in low or middle-income countries. Evidence on the role of exercise and physical activity is emerging in studies conducted in Africa, specifically Nigeria, Zimbabwe and South Africa [55–57]. For instance, Ezema [58] reported improvements in cardiopulmonary fitness and immune status among aerobic exercisers versus non-exercisers with HIV in Nigeria. Mkandla [59] reported improvements in quality of life for people living with HIV and neuropathy engaging in twice weekly PRE in Zimbabwe. Further evidence from South Africa included evaluating the impact of home-based and pedometer walking interventions as a way to enhance physical activity for adults living with HIV [60, 61], highlighting the role of exercise beyond the context of high income countries.

Results of this review align with results of systematic reviews focused on aerobic exercise [12, 62]. Our review included studies with exercise frequency of three times per week, however “results are consistent with a systematic review that assessed the effect of combined twice weekly aerobic and resistive exercise on cardiorespiratory status, quality of life, physiologic and functional outcomes concluding that exercise is safe and beneficial for medically stable adults living with HIV” [63, 64] [12]. Gomes-Neto et al. reported that aerobic exercise is beneficial for cardiorespiratory health, body composition and quality of life. In addition, Leach and colleagues conducted a systematic review assessing the effect of twice weekly aerobic or resistive exercise on body composition. Individual results from the four included studies reported increases in body mass, sum of skinfolds and limb girth [65]. In the aforementioned reviews, meta-analyses were not performed. Gomes-Neto conducted a systematic review and meta-analysis comparing combined PRE and aerobic exercise conducted at least twice per week for 4 weeks. They included seven studies and conducted meta-analyses that demonstrated benefits to VO2max, muscle strength and quality of life [53]. Another systematic review and meta-analysis that investigated the effect of at least twice weekly PRE and aerobic exercise on body composition and metabolic outcomes found that exercise resulted in reductions in body mass index, body fat percentage, triceps skinfold thickness, waist circumference and waist-to-hip ratio [66]. While our inclusion criteria were more strict than these reviews (thrice versus twice weekly exercise), our systematic review included a greater number of studies, sub-group and meta-analyses providing further detail on the pooled effect of exercise and co-interventions with exercise for adults living with HIV. Our results are consistent with four more recently published randomized controlled trials involving thrice weekly PRE with adults living with HIV. Authors reported similar improvements with PRE (or combined PRE and aerobic exercise) for outcomes of cardiorespiratory health, body composition, strength, quality of life, and CD4 count consistent with the results of this review [67–70].

Evidence is emerging documenting the benefits of exercise and physical activity on neurocognitive function for adults with HIV showing positive correlations between physical activity and cognition [71, 72], and cardiopulmonary fitness and cognition [73]. One study demonstrated higher levels of executive function among self-reported physical activity compared with sedentary adults with HIV, with larger benefits associated with greater intensity and duration of physical activity, suggesting the more one exercises the greater the benefits [74]. We did not assess cognitive outcomes, because this was outside the scope of our review; however the effect of exercise on cognitive health is increasingly important to consider as adults age with HIV.

The effects of exercise on metabolic outcomes were considered more frequently in studies published in the post combination antiretroviral therapy era. Eleven of the included studies assessed metabolic outcomes [25, 26, 28–30, 33, 34, 37–39, 42] of which eight reported improvements with PRE (or combined PRE and aerobic exercise) including increases in high density lipoprotein (HDL) cholesterol, and decreases in total cholesterol, triglycerides, fasting insulin, and C-Reactive Protein (CRP) [25, 28, 29, 33, 34, 38, 39, 42]. As the field of literature expands, future updates of this review should assess the impact of exercise co-interventions, the impact of exercise on outcomes including metabolic and inflammatory markers and make an attempt to conduct sub-group analyses that acknowledge changing health-related consequences that may occur in the pre versus post era of combination antiretroviral therapy.

Collectively, the concept of ‘exercise as medicine’ is gaining momentum in chronic disease, emphasizing the importance of exercise as a self-management strategy for adults living with HIV [9, 75]. However, few people with HIV exercise regularly [76]. Individuals living with HIV multi-morbidity may exhibit a range of readiness to exercise [77]. Hence there is a need to consider innovative ways to enhance physical activity among people with HIV specifically suited to the individual. Strategies to enhance self-management and the uptake of physical activity may include personalized text messaging interventions and physical activity monitors [78–80]. Health providers should consider the different perceptions of terms such as ‘exercise’ versus ‘physical activity’ among people living with HIV. For instance, physical activity may be just as effective as regular exercise showing similar benefits to cardiorespiratory status [57]. Hence, health providers might provide further choice to their clients if physical activity is just as beneficial as more traditional forms of exercise.

### Implications for research

Evidence for the safety and effectiveness of exercise is increasing with mounting reviews, clinical guidelines, and evidence-informed recommendations that support exercise interventions for adults living with HIV [63, 64, 66, 81–86], and specifically the benefits of exercise for older adults living with HIV and multi-morbidity [52, 87, 88].

Similarly reported in our previous review, “interpretations of weight and body composition outcomes should be considered relative to the publication dates of the included studies. Prior to the advent of combination antiretroviral therapy, studies on exercise tended to include participants with AIDS-wasting, whereas recent evidence includes participants on combination antiretroviral therapy with lipodystrophy, body fat redistribution, or hyperinsulinemia” [12]. Furthermore, we found more published studies that assessed interventions such as tai chi [89, 90] or co-interventions with exercise such as diet and/or nutritional counselling [25, 26, 30, 91] metformin [28, 38], creatine [32] and pioglitazone [34]. Finally, this review included only 23% of women participants revealing an under-representation of women in the HIV and exercise literature.

As individuals live longer and age with HIV, future research should involve older adults and people living with multi-morbidity such as liver, cardiovascular, kidney, and bone and joint diseases [4]. Finally, given most of the studies included in the review involved exercise interventions in clinical settings that were supervised by health or research personnel, future research studies might consider evaluating the impact of non-supervised or community-based exercise interventions to reflect a self-management model for people with HIV [92].

## Conclusions

Engaging in progressive resistive exercise, or a combination of PRE and aerobic exercise three times per week for at least six weeks appears safe and can lead to significant improvements cardiorespiratory fitness (maximum oxygen consumption, exercise time), strength (chest press, knee flexion), weight, and body composition (leg muscle area, arm and thigh girth). Greater improvements in weight and body composition were found with resistive exercise compared to aerobic interventions. Interpreting weight and body composition changes should be considered relative to the introduction of combination antiretroviral therapy. Progressive resistive exercise is safe and beneficial for medically stable adults living with HIV.

## Additional files

Additional file 1: Search Strategy Example for the Progressive Resistive Exercise and HIV Systematic Review Update. (PDF 83 kb)

Additional file 2: Detailed Characteristics of Included Studies in the Progressive Resistive Exercise (PRE) and HIV Systematic Review (*n* = 20 studies). (PDF 248 kb)

Additional file 3: Details of Outcomes and Authors’ Conclusions of Individual Studies Included in the Progressive Resistive Exercise (PRE) and HIV Systematic Review (*n* = 20 studies) (PDF 205 kb)

Additional file 4: GRADE Summary of Finding Table: Progressive Resistive Exercise (PRE) or Combined Progressive Resistive Exercise (PRE) and Aerobic Exercise Compared with No Exercise for adults living with HIV (PDF 111 kb)

## Abbreviations

1-RM: 1 repetition maximum
BMI: Body mass index
bpm: Beats per minute
GRADE: Grading of Recommendations Assessment, Development, and Evaluation
HIV: Human immunodeficiency virus
PRE: Progressive resistive exercise
