# Supplementary material for: Effectiveness of Progressive Resistive Exercise (PRE) in the context of HIV: systematic review and meta-analysis using the Cochrane Collaboration protocol
Source: BMC Infect Dis. 2017 Apr 12;17:268. doi: 10.1186/s12879-017-2342-8 (PMC5389006; doi:10.1186/s12879-017-2342-8)
Supplement: Supplementary file 1 — Search Strategy Example for the Progressive Resistive Exercise and HIV Systematic Review Update. (PDF 83 kb) [file 12879_2017_2342_MOESM1_ESM.pdf]

Additional File 1 – Search Strategy Example for the Progressive Resistive Exercise and HIV Systematic Review Update

Database: Ovid MEDLINE(R) <1946 to April Week 2 2013>, Ovid MEDLINE(R) In-Process & Other Non-Indexed Citations <April 19,2013>

Search Strategy:

----

1. exp HIV/ (78312)
2. exp HIV Infections/ (217795)
3. exp Anti-HIV Agents/ (48954)
4. Antiretroviral Therapy, Highly Active/ (15552)
5. exp HIV Long-Term Survivors/ (520)
6. (hiv or human immunodeficiency or acquired immunodeficiency syndrome or acquired immune deficiency syndrome or aids or hiv+).tw. (291200)
7. or/1-6 (328909)
8. limit 7 to aids (307770)
9. Physical Exertion/ (51674)
10. Physical Fitness/ (20556)
11. exp Sports/ (103313)
12. "Physical Education and Training"/ (11404)
13. exp Exercise Therapy/ (27149)
14. Resistance Training/ (2449)
15. Weight Lifting/ (3717)
16. exp Exercise/ (101134)
17. "Anaerobic exercise".tw. (324)
18. "progressive resistance".tw. (551)
19. "progressive resistive".tw. (66)
20. "resistance exercise".tw. (2275)
21. (resistance adj training).tw. (3133)
22. (strength adj training).tw. (2546)
23. ("weight bearing" adj exercise).tw. (355)
24. "weight lifting".tw. (547)
25. "weight training".tw. (719)
26. (Isometric adj3 exercise*).tw. (2099)
27. (Isometric adj2 strengthening).tw. (27)
28. (Isotonic adj3 exercise*).tw. (286)
29. (Isotonic adj2 strengthening).tw. (4)
30. (resistant adj3 exercise).tw. (44)
31. "resistant training".tw. (2)
32. exercise.tw. (162141)
33. or/9-32 (308001)
34. 8 and 33 (1158)
35. randomized controlled trial.pt. (347205)
36. controlled clinical trial.pt. (85774)
37. randomized.ab. (265085)

38. placebo.ab. (143408)
39. drug therapy.fs. (1601703)
40. randomly.ab. (192990)
41. trial.ab. (273734)
42. groups.ab. (1245205)
43. or/35-42 (3103475)
44. 34 and 43 (458)
45. exp animals/ not humans.sh. (3802339)
46. 44 not 45 (450)
47. limit 46 to yr="2006 -Current" (174)
48. remove duplicates from 47 (166)
49. limit 48 to "all child (0 to 18 years)" (29)
50. limit 49 to "all adult (19 plus years)" (21)
51. 49 not 50 (8)
52. 48 not 51 (158)

NOTE: Lines 35-44 comprise Box 6.4.c: Cochrane Highly Sensitive Search Strategy for identifying randomized trials in MEDLINE: sensitivity-maximizing version (2008 revision); Ovid format) from:

Lefebvre C, Manheimer E, Glanville J. Chapter 6: searching for studies. In: Higgins JPT, Green S, editors. Cochrane handbook for systematic reviews of interventions. Version 5.0.2 [updated September 2009]. The Cochrane Collaboration, 2009

NOTE: search strategy was modified accordingly for the other databases.
