# Supplementary material for: Effectiveness of Progressive Resistive Exercise (PRE) in the context of HIV: systematic review and meta-analysis using the Cochrane Collaboration protocol
Source: BMC Infect Dis. 2017 Apr 12;17:268. doi: 10.1186/s12879-017-2342-8 (PMC5389006; doi:10.1186/s12879-017-2342-8)
Supplement: Supplementary file 2 — Detailed Characteristics of Included Studies in the Progressive Resistive Exercise (PRE) and HIV Systematic Review (n = 20 studies). (PDF 248 kb) [file 12879_2017_2342_MOESM2_ESM.pdf]

Additional File 2: Detailed Characteristics of Included Studies in the Progressive Resistive Exercise (PRE) and HIV Systematic Review (n=20 studies)

Study	Methods	Sample Size (at baseline)	% Women	% taking combination ART	Participants (at study completion)	Withdrawal Rate	Intervention	Duration and Frequency	Location of Exercise	Supervision
Agin (2001)	Randomized combined PRE plus whey protein, whey protein only versus PRE only [3 groups]<br><br>PRE ONLY VERSUS PRE+WHEY VERSUS WHEY ONLY	43 (with wasting)	100%	Unknown (recruitment was conducted just following availability of HAART)	30	13/43 (30%)	PRE EXERCISE:<br>*PRE*: Resistance training of seven major muscle groups were performed; 3 sets of 10 exercises @ 8-10 repetitions per/set as per ACSM guidelines; Week 1: loads were 50% of baseline 1-RM. Loads increased approximately 75% of 1-RM with adjustments based on number of repetitions and percentages of 1-RM. Loads were increased by at least 2.5 pounds when a participant completed 10 consecutive reps for a muscle group without fatigue.<br><br>WHEY PROTEIN:<br>1g/kg of whey protein powder for 14 weeks.	Duration unknown; 3 times per week for 14 weeks	Hospital	Supervised

Additional File 2: Detailed Characteristics of Included Studies in the Progressive Resistive Exercise (PRE) and HIV Systematic Review (n=20 studies)

Study	Methods	Sample Size (at baseline)	% Women	% taking combination ART	Participants (at study completion)	Withdrawal Rate	Intervention	Duration and Frequency	Location of Exercise	Supervision
Agostini (2009)* #	Randomized combined AER+PRE versus diet and aerobic exercise recommendation alone (no exercise) [2 groups]<br><br>CONSTANT AEROBIC + PRE + DIET VERSUS DIET and EXERCISE RECOMMENDATION ONLY	76	39%	100%	70	6/76 (8%)	<u>EXERCISE (PRE+AER) + CONTROLLED DIET INTERVENTION GROUP</u>: Participants placed on a systematic and controlled diet and physical exercisers (aerobic activity of moderate intensity). *Aerobic components included:* walking on a treadmill for 40 minutes, run 30 minutes and stair climb for 15 minutes. *Anaerobic components included*: 40 minutes of PRE weight training in arms and legs; 10 cycles 3 repetitions. Weight training included 2kg for women and 5 kg for men. Cool Down and Relaxation: 5 minutes. Intensity: Medium intensity	70 minutes; 3X per week for 48 weeks	NR	NR

Additional File 2: Detailed Characteristics of Included Studies in the Progressive Resistive Exercise (PRE) and HIV Systematic Review (n=20 studies)

Study	Methods	Sample Size (at baseline)	% Women	% taking combination ART	Participants (at study completion)	Withdrawal Rate	Intervention	Duration and Frequency	Location of Exercise	Supervision
Agostini (2009)* # (continued)	CONSTANT AEROBIC + PRE + DIET VERSUS DIET and EXERCISE RECOMMENDATION ONLY						<u>DIET and AEROBIC EXERCISE RECOMMEND GROUP (CONTROL):</u> Participants were given advice to follow a standard diet and physical exercise plan according to current recommendations.			
Balasubramanyam (2011)* #	Randomized trial with five comparison groups<br><br>1) DIET+ EXERCISE (lifestyle change) plus 2 placebos versus<br>2) DIET + EXERICSE combined with niacin and fenofibrate versus<br>3) Diet and Exercise + niacin only plus 1 placebo versus<br>4) Diet and Exercise + fenofibrate only plus 1 placebo versus<br>5) Usual Care (with 2 placebos). [5 groups]<br>**Note for this systematic review we compared Group 1 (exercise + diet) to Group 5 (usual care).*	191 (with dyslipidemia)	13%	100%	128 (68 participants of in Group 1 and 5)	63/191 (33%)	<u>DIET + EXERCISE INTERVENTION GROUP</u>: *Diet Intervention*: Participants were taught a weight-maintaining diet. *Exercise Intervention*: Participants engaged in an exercise program following ACSM guidelines. *AEROBIC Component*: Participants began with 10 minutes stretching and 5 minutes warm-up; followed by 20-25 minutes of aerobic exercises (stationary bike and	75-90 minutes; 3X per week for 24 weeks	Study gym	Supervised

Additional File 2: Detailed Characteristics of Included Studies in the Progressive Resistive Exercise (PRE) and HIV Systematic Review (n=20 studies)

Study	Methods	Sample Size (at baseline)	% Women	% taking combination ART	Participants (at study completion)	Withdrawal Rate	Intervention	Duration and Frequency	Location of Exercise	Supervision
Balasubramanyam (2011)* # (continued)	CONSTANT AEROBIC + PRE + DIET VERSUS DIET and EXERCISE RECOMMENDATION ONLY						ergometer) at intensity of 70-85% maximal heart rate or 60-80% HR reserve, followed by 5-10 minute cool down period. Intensity measured using the modified Borg Rate of Perceived Exertion (RPE) scale. *PRE*: Resistive exercises were performed for 45-50 minutes; three sets of 8-12 repetitions with a rest break of 1-3 minutes between each set; followed by 5-10 minutes cool down. Intensity: 60-80% 1 repetition maximum (1RM) of leg and bench press. After a given weight was lifted 8-12 times until muscular failure (unable to complete additional repetitions).			

Additional File 2: Detailed Characteristics of Included Studies in the Progressive Resistive Exercise (PRE) and HIV Systematic Review (n=20 studies)

Study	Methods	Sample Size (at baseline)	% Women	% taking combination ART	Participants (at study completion)	Withdrawal Rate	Intervention	Duration and Frequency	Location of Exercise	Supervision
Balasubramanyam (2011)* # (continued)	CONSTANT AEROBIC + PRE + DIET VERSUS DIET and EXERCISE RECOMMENDATION ONLY						Study trainers provided exercise plans to participants in this alternate program and reviewed their progress biweekly.<br><br><u>DIET AND EXERCISE RECOMMENDATION ONLY (USUAL CARE)</u>: Participants received general advice on a heart healthy diet, kept a 7 day food record and received feedback on their caloric intake during a single baseline visit. Participants received a copy of "The Activity Pyramid" recommended by ACSM.			

Additional File 2: Detailed Characteristics of Included Studies in the Progressive Resistive Exercise (PRE) and HIV Systematic Review (n=20 studies)

Study	Methods	Sample Size (at baseline)	% Women	% taking combination ART	Participants (at study completion)	Withdrawal Rate	Intervention	Duration and Frequency	Location of Exercise	Supervision
Bhasin (2000)	Randomized trial with four comparison groups<br><br>1) EXERCISE + TESTOSTERONE versus<br>2) TESTOSTEERONE ONLY versus<br>3) EXERCISE only (with placebo) versus<br>4) NON-EXERCISING CONTROL (with placebo)<br>[4 groups]<br><br>PRE versus PRE+TESTOSTERONE versus TESTOSTERONE ONLY versus NON-EXERCISING CONTROL	61 (with involuntary weight loss and low testosterone levels)	0%	100% taking ARVs (unclear whether it was cART)	49	12/61 (20%)	PRE INTERVENTION:<br>*PRE*: Resistance training of 5 upper and lower body exercises. Week 1-4: High volume (3 sets of 12-15 repetitions), low intensity (60% of initial 1-RM) resistance exercise. Week 5-10: Progressive periodic high intensity (90% of 1-RM on heavy days, 80% on medium days, and 70% on light days), low volume (4 sets of 4-6 repetitions each). Week 11-16: Resistance loads were increased by 7% for upper body and 12% for lower body exercises; number of sets increased to 5.<br>TESTOSTERONE INTERVENTION:<br>Intramuscular injections of 100mg/week of testosterone.	Duration unknown; 3X per week for 16 weeks	NR	NR

Additional File 2: Detailed Characteristics of Included Studies in the Progressive Resistive Exercise (PRE) and HIV Systematic Review (n=20 studies)

Study	Methods	Sample Size (at baseline)	% Women	% taking combination ART	Participants (at study completion)	Withdrawal Rate	Intervention	Duration and Frequency	Location of Exercise	Supervision
Dolan (2006) #	Randomized exercise and control groups [2 groups]<br><br>CONSTANT AEROBIC + PRE VERSUS NON-EXERCISING CONTROL	40 (with self-reported and physical evidence of changes in fat distribution)	100%	82% taking ARVs (unclear whether it was cART)	38	2/40 (5%)	INTERVENTION GROUP (Aerobic + PRE Exercise): Combined PRE and aerobic exercise for 2 hours total. *Aerobic Component*: 5 minute warm-up on stationary bike at 50% estimated HRmax, followed by standard flexibility routine and aerobic and PRE exercise according to ACSM guidelines followed by a cool down period. *PRE Component*: concentric and eccentric phases of 6 selected upper and lower body muscle groups; Week 1: 3 sets of 10 reps for each muscle group at 60% 1-RM, 3-5 seconds between reps rest, 2 min rest between sets, 4 min rest between muscle groups; week 3-16: 4 sets of 8 reps for each	2 hours, 3X per week for 16 weeks	Home	Supervised

Additional File 2: Detailed Characteristics of Included Studies in the Progressive Resistive Exercise (PRE) and HIV Systematic Review (n=20 studies)

Study	Methods	Sample Size (at baseline)	% Women	% taking combination ART	Participants (at study completion)	Withdrawal Rate	Intervention	Duration and Frequency	Location of Exercise	Supervision
Dolan (2006) # (continued)	CONSTANT AEROBIC + PRE VERSUS NON-EXERCISING CONTROL						muscle group at 70% 1-RM (Week 2-3), and 80% 1-RM (week 4-16), 2-3 seconds between reps rest, 1 min rest between sets, 2 min rest between muscle groups. Each repetition lasted 6-10 seconds each.<br><br>NON-EXERCISING CONTROL GROUP: Usual care			
Driscoll (2004a) #	Randomized combined exercise and metformin and metformin-only group [2 groups]<br><br>CONSTANT AEROBIC + PRE + METFORMIN VERSUS METFORMIN ONLY	37 (evidence of fat redistribution and hyperinsulinemia)	20%	100%	25	12/37 (32%)	INTERVENTION GROUP (Exercise + Metformin): Constant aerobic exercise followed by resistive training consisting of 20 minutes aerobic exercise on stationary cycle at 60% HRmax (week 1-2) and progressing to 30 minutes at 75% HRmax (week 3-12) according to ACSM guidelines, 5min warm-up on stationary bike, standard flexibility	20-30 minute (aerobic) plus unknown total minutes (PRE); 3X per week for 12 weeks.	Hospital	Supervised

Additional File 2: Detailed Characteristics of Included Studies in the Progressive Resistive Exercise (PRE) and HIV Systematic Review (n=20 studies)

Study	Methods	Sample Size (at baseline)	% Women	% taking combination ART	Participants (at study completion)	Withdrawal Rate	Intervention	Duration and Frequency	Location of Exercise	Supervision
Driscoll (2004a) # (continued)	CONSTANT AEROBIC + PRE + METFORMIN VERSUS METFORMIN ONLY						routine, followed by resistance training.<br>*PRE* consisted of 3 sets of 10 repetitions for every muscle group, resting 2-3 seconds between repetitions, 2 minutes between sets, and 4 minutes between muscle group. Week 1: initial intensity of PRE was 60% 1-RM; week 2-4 intensity increased to 70% 1-RM; week 4-12 intensity of 80% 1-RM. 1-RM was measured every other week and load adjusted to maintain relative intensity at 80% 1-RM.<br><br><u>METFORMIN ONLY GROUP</u>: 500 mg of metformin twice per day, with a dose increase to 850 mg twice a day (week 2-12).			

Additional File 2: Detailed Characteristics of Included Studies in the Progressive Resistive Exercise (PRE) and HIV Systematic Review (n=20 studies)

Study	Methods	Sample Size (at baseline)	% Women	% taking combination ART	Participants (at study completion)	Withdrawal Rate	Intervention	Duration and Frequency	Location of Exercise	Supervision
Farinatti (2010)* #	Randomized exercise and control groups [2 groups]<br><br>CONSTANT AEROBIC + PRE VERSUS NON-EXERCISING CONTROL	27	Not reported	100%	27	0/27 (0%)	INTERVENTION GROUP (Aerobic + PRE Exercise): 90 minute total. *Constant Aerobic Exercise*: Cyclo-ergometer for 30 min at moderate intensity. *PRE Strength training*: 3 sets of 12 reps of 5 exs at 60-80% 12-RM. 1st week - 3 sets of 21 reps at 60% 12 rep maximum (12 RM) for all exercises. For remaining weeks, 80% of 12-RM for the following: leg press, bench press, knee extension, seated bilat row, abdominal sit-ups with rest intervals of 2-3 min between sets and exercises. *Flexibility*: 10 minutes - 2 sets of 30 sec at max range of motion of 8 exercises (involving all major joints). NON-EXERCISING CONTROL GROUP: No intervention.	90 minutes; 3X per week for 12 weeks	NR	Supervised

Additional File 2: Detailed Characteristics of Included Studies in the Progressive Resistive Exercise (PRE) and HIV Systematic Review (n=20 studies)

Study	Methods	Sample Size (at baseline)	% Women	% taking combination ART	Participants (at study completion)	Withdrawal Rate	Intervention	Duration and Frequency	Location of Exercise	Supervision
Fitch (2012)*#	Randomized trial: 1) exercise (lifestyle modification - LSM) and placebo (EXERCISE ONLY) versus 2) exercise (lifestyle modification) + metformin (EXERCISE+METFORMIN) versus 3) no LSM and metformin only (METFORMIN ONLY) versus 4) CONTROL (no LSM and placebo) [4 groups]<br><br>CONSTANT AEROBIC + PRE VERSUS NON-EXERCISING CONTROL<br><br>CONSTANT AEROBIC + PRE + METFORMIN VERSUS METFORMIN ONLY	50 (with metabolic syndrome)	24%	100%	36	14/50 (28%)	<u>INTERVENTION GROUP - EXERCISE (LIFESTYLE MODIFICATION):</u> Included exercise 3 times per week with dietary counselling once per week.<br>*Aerobic*: Warm up was 5 min of stationary bike at 50% max HR. Aerobic training performed using a stationary cycle - participant exercised for 20 min at 60% their maximal HR for the first 2 weeks followed by 30 min at 75% their maximal HR for the study duration. [20 min total]<br>*PRE*: Aerobic training followed by 30 min of PRE. Exercises included: leg press, chest press, knee extension, lateral pull down, knee flexion, and triceps dip. Participants	50 minutes total (20 aerobic; 30 PRE); 3X per week for 52 weeks	NR	Supervised

Additional File 2: Detailed Characteristics of Included Studies in the Progressive Resistive Exercise (PRE) and HIV Systematic Review (n=20 studies)

Study	Methods	Sample Size (at baseline)	% Women	% taking combination ART	Participants (at study completion)	Withdrawal Rate	Intervention	Duration and Frequency	Location of Exercise	Supervision
Fitch (2012)* # (continued)	CONSTANT AEROBIC + PRE VERSUS NON-EXERCISING CONTROL<br><br>CONSTANT AEROBIC + PRE + METFORMIN VERSUS METFORMIN ONLY						performed 3 sets of 10 reps for each exercise, effort increased over 6 months from 60% to 80% of 1RM. For those unable to reach 80% 1RM the resistance was increased as tolerated. [30 min total]<br>*Dietary Counseling*: Investigators covered curriculum modelled after a diabetes prevention program. Initial sessions were completed within the first 18 weeks with review and reinforcement for the remainder of the study.<br><br><u>EXERCISE (LSM) + METFORMIN GROUP</u>: Exercise (or LSM) as above plus 500 mg of metformin twice a day with a dose increase to 850 mg twice a day after 3 months.			

Additional File 2: Detailed Characteristics of Included Studies in the Progressive Resistive Exercise (PRE) and HIV Systematic Review (n=20 studies)

Study	Methods	Sample Size (at baseline)	% Women	% taking combination ART	Participants (at study completion)	Withdrawal Rate	Intervention	Duration and Frequency	Location of Exercise	Supervision
Grinspoon (2000) #	Randomized trial with 4 comparison groups: 1) PRE+AEROBIC versus 2) PRE+AEROBIC + Testosterone versus 3) Testosterone only versus 4) Control [4 groups]<br><br>CONSTANT AEROBIC + PRE VERSUS NON-EXERCISING CONTROL	54 (with AIDS-related wasting)	0%	72%	43	11/54 (20%) [4/26 (15%) from the 2 comparison groups of interest]	INTERVENTION EXERCISE GROUP: Supervised progressive strength training and constant aerobic training. *Aerobic*: 20 min on stationary cycle at 60-70% HRmax, 15min cool-down followed by resistance training. *PRE*: performed isotonically on computerized equipment and included: leg extension, leg curl, leg press, latissimus doris pull-down, arm curl, and triceps extension. 1-RM weight was established at baseline. Participants increased resistance: weeks 1 and 2, 2 sets at 8 reps per set, 60% 1-RM; weeks 3 to 6, 2 sets, 8 reps per set, 70% 1-RM; weeks 7 to 12, 3 sets, 8 reps per set, 80% 1-RM.	Total exercise time unknown (20 aerobic+15 cool-down + PRE unknown) 3x per week for 12 weeks.	NR	Supervised

Additional File 2: Detailed Characteristics of Included Studies in the Progressive Resistive Exercise (PRE) and HIV Systematic Review (n=20 studies)

Study	Methods	Sample Size (at baseline)	% Women	% taking combination ART	Participants (at study completion)	Withdrawal Rate	Intervention	Duration and Frequency	Location of Exercise	Supervision
Lindegaard (2008)* #	Randomized trial of aerobic versus progressive resistive exercise [2 groups]<br><br>INTERVAL AEROBIC VERSUS PRE	20 (with dyslipidemia, lipodystrophy)	0%	100%	18	2/20 (10%)	AEROBIC EXERCISE GROUP: Aerobic exercise consisted of 8 programs with 35 min of interval training. 5 min warm-up. Intensity varied from 50-100% VO2max. For the first 8 weeks the mean intensity was targeted at 65% VO2max and the last 8 weeks were targeted to 75% of VO2max.<br><br>PRE (RESISTANCE) EXERCISE GROUP: PRE consisted of 8 exercises (leg curl, pull down, seated leg press, chest press, seated rows, leg extension, abdominal crunch and back extension) in resistance training machines for 45-60 min. The # of reps and sets changed every week and the resting interval was 60-120 sec. 1-RM calculated as 100% of 3-RM results.	Aerobic Session (35 minutes); PRE Session (45-60 minutes); 3X per week for 16 weeks	Public Fitness Centre	Supervised

Additional File 2: Detailed Characteristics of Included Studies in the Progressive Resistive Exercise (PRE) and HIV Systematic Review (n=20 studies)

Study	Methods	Sample Size (at baseline)	% Women	% taking combination ART	Participants (at study completion)	Withdrawal Rate	Intervention	Duration and Frequency	Location of Exercise	Supervision
Lox (1995) #	Randomized to two exercise groups (PRE and aerobic) and one control group [3 groups]<br><br>CONSTANT AEROBIC VERSUS PRE VERSUS NON-EXERCISING CONTROL~	22 (aerobic and control groups only)	0%	100% (taking some form of ARV therapy that may or may not have been in combination)	21	1/22 (4%)	INTERVENTION GROUP (AEROBIC): Stationary bike, 45 minutes total: 5 minutes warm-up (stretching), 24 minutes cycle ergometer at 50-60% heart rate reserve (HRR), 15 minutes cool-down.<br><br>INTERVENTION GROUP (PRE): 45 minutes total. Isotonic resistance to major muscle groups in legs, arms and upper body. Resistance was initiated at 60% of an individual's 1-RM and increased by either 5 or 10 pounds at a time after successful performing 3 sets of 10 reps at constant weight.	45 minutes total; 3X per week for 12 weeks	NR	Supervised

Additional File 2: Detailed Characteristics of Included Studies in the Progressive Resistive Exercise (PRE) and HIV Systematic Review (n=20 studies)

Study	Methods	Sample Size (at baseline)	% Women	% taking combination ART	Participants (at study completion)	Withdrawal Rate	Intervention	Duration and Frequency	Location of Exercise	Supervision
Ogalha (2011)*	Randomized exercise and control group [2 groups]<br><br>AEROBIC + PRE + NUTRITION COUNSELING VERSUS NUTRITION COUNSELING ALONE (CONTROL)	70 (lipodystrophy in 54% of participants)	46%	100%	63	7/70 (10%)	EXERCISE + NUTRITIONAL COUNSELING (INTERVENTION) GROUP: 1 hour gym class 3 times per week plus monthly dietary counseling. Intensity was 75% maximum HR<br><br>NUTRITIONAL COUNSELING (MONTHLY) NON-EXERCISING CONTROL GROUP: Monthly dietary counselling by a nutrition specialist. Counseling sessions included 50 minute discussion on dietary needs and recommendations. Participants also received a 30 min orientation on the importance of regular physical activity and how to include it in daily routine. Stimulated to perform activities (running, biking or walking) for 1 hour at least 3 times per week.	3X per week for 24 weeks	Fitness centre	Supervised

Additional File 2: Detailed Characteristics of Included Studies in the Progressive Resistive Exercise (PRE) and HIV Systematic Review (n=20 studies)

Study	Methods	Sample Size (at baseline)	% Women	% taking combination ART	Participants (at study completion)	Withdrawal Rate	Intervention	Duration and Frequency	Location of Exercise	Supervision
Perez-Moreno (2007)* #	Randomized exercise and control groups [2 groups]<br><br>CONSTANT AEROBIC + PRE VERSUS NON-EXERCISING CONTROL	27 (prison inmates living with Hepatitis C co-infection)	0%	10%	19	8/27 (30%	<u>EXERCISE (AEROBIC + PRE) INTERVENTION GROUP:</u> 3 weekly sessions of 90-min duration each. Each session started and ended with a 10-min warm-up and cool-down period, respectively, consisting of cycle ergometer pedalling at very light workloads and stretching exercises for all major muscle groups. The 70-min core portion of the training session was divided into resistance and aerobic training.<br><br>*PRE*: Resistance training included 11 exercises engaging 11 major muscle groups. *Stretching exercises* involved an exercise performed at the end of each set of resistance exercise. In month 1, participants	135 minutes total (PRE+ Aerobic plus warm up and cool-down); 3X per week for 16 weeks.	Prison	Supervised

Additional File 2: Detailed Characteristics of Included Studies in the Progressive Resistive Exercise (PRE) and HIV Systematic Review (n=20 studies)

Study	Methods	Sample Size (at baseline)	% Women	% taking combination ART	Participants (at study completion)	Withdrawal Rate	Intervention	Duration and Frequency	Location of Exercise	Supervision
Perez-Moreno (2007)* # (continued)	CONSTANT AEROBIC + PRE VERSUS NON-EXERCISING CONTROL						performed two and one set of exercises for large and small muscle groups and all sets were performed at a resistance that allowed 12–15 repetitions. Then, the resistance used was individually adjusted to allow the completion of 8–10 repetitions for three sets of the large muscle group exercises and two sets of the small muscle group exercises. The resistance used for each exercise was increased by 5–10% when the participant could perform the prescribed maximal repetitions per set. After an increase in resistance, the repetitions per set typically decreased to the low end of the prescribed repetition range (12 or 8			

Additional File 2: Detailed Characteristics of Included Studies in the Progressive Resistive Exercise (PRE) and HIV Systematic Review (n=20 studies)

Study	Methods	Sample Size (at baseline)	% Women	% taking combination ART	Participants (at study completion)	Withdrawal Rate	Intervention	Duration and Frequency	Location of Exercise	Supervision
Perez-Moreno (2007)* # (continued)	CONSTANT AEROBIC + PRE VERSUS NON-EXERCISING CONTROL						repetitions). Abdominal crunches and low back extensions were performed in two sets of 15–20 repetitions at the start of the program and in three sets of 20 repetitions at the end of it.<br><br>*Aerobic Exercise:* At the beginning of the program, aerobic training consisted of pedalling on a cycle ergometer for 20 min at 70% of the age-predicted maximum heart rate. The duration and intensity of the sessions were gradually increased during the 4-month period so that participants completed 45 min of continuous pedalling at 80% of HRmax by the end of the training program. For			

Additional File 2: Detailed Characteristics of Included Studies in the Progressive Resistive Exercise (PRE) and HIV Systematic Review (n=20 studies)

Study	Methods	Sample Size (at baseline)	% Women	% taking combination ART	Participants (at study completion)	Withdrawal Rate	Intervention	Duration and Frequency	Location of Exercise	Supervision
Perez-Moreno (2007)* # (continued)	CONSTANT AEROBIC + PRE VERSUS NON-EXERCISING CONTROL						participants in the poorest physical condition, it was sometimes necessary to divide the first sessions into shorter time intervals to complete the total 20-min target duration.<br><br><u>NON-EXERCISING CONTROL GROUP</u>: Participants followed their usual sedentary lifestyle (physical activity level < 2; walking for a total of 30-60 minutes three days per week) and performing no strenuous exercise such as running, cycling, swimming or resistance training.			

Additional File 2: Detailed Characteristics of Included Studies in the Progressive Resistive Exercise (PRE) and HIV Systematic Review (n=20 studies)

Study	Methods	Sample Size (at baseline)	% Women	% taking combination ART	Participants (at study completion)	Withdrawal Rate	Intervention	Duration and Frequency	Location of Exercise	Supervision
Rigbsy (1992)	Randomized exercise and control (counselling) groups [2 groups]<br><br>CONSTANT AEROBIC + PRE VERSUS NON-EXERCISING CONTROL	45 (37 HIV+)	0%	Not reported	31 (24 HIV+)	13/37 (35%)	INTERVENTION GROUP: Stationary bike 60 minutes total @60-80% HRreserve x 20 min (2 min warm-up and 3 min cool down at low intensity.) Stretching x 10-15 minutes. Strengthening x 20-25 minutes.<br><br>NON-EXERCISING CONTROL GROUP: Received 90-120 minutes of counselling 1-2 times per week for 12 weeks.	90-100 minutes; 3 x/ week for 12 weeks	NR	Supervised

Additional File 2: Detailed Characteristics of Included Studies in the Progressive Resistive Exercise (PRE) and HIV Systematic Review (n=20 studies)

Study	Methods	Sample Size (at baseline)	% Women	% taking combination ART	Participants (at study completion)	Withdrawal Rate	Intervention	Duration and Frequency	Location of Exercise	Supervision
Sakkas (2009)*	Randomized exercise and control groups [2 groups]<br><br>PRE + CREATINE VERSUS PRE ONLY	40	0%	75%	33	7/40 (18%)	<u>PRE+CREATINE GROUP:</u><br>*PRE:* Performed using a multi-gym fitness system. Included ankle dorsiflexion, ankle plantarflexion, ankle plantarflexion with bent knee, leg press, leg curls, pectoralis chest exercises, triceps pushdown, biceps curls, and 3 types of abdominal crunches. Each session included 4 sets of 8 reps at 80% of 1-RM for each exercise. Progression of Intensity: 1-RM for each exercise was reassessed once every 2 weeks and training intensity adjusted to maintain 80% of 1-RM.<br><br>*Creatine*: Participants began with loading doses of 20g/day (divided among 4 doses) or	90 minutes; 3x per week for 12 weeks	NR	NR

Additional File 2: Detailed Characteristics of Included Studies in the Progressive Resistive Exercise (PRE) and HIV Systematic Review (n=20 studies)

Study	Methods	Sample Size (at baseline)	% Women	% taking combination ART	Participants (at study completion)	Withdrawal Rate	Intervention	Duration and Frequency	Location of Exercise	Supervision
Sakkas (2009)* (continued)	PRE + CREATINE VERSUS PRE ONLY						an equivalent number of placebo capsules for 5 days followed by maintenance dosing of 4.8g/day.<br><br>PRE ONLY GROUP: Participants received placebo for Creatine. PRE as above.			
Sattler (1999)	Randomized combined exercise plus testosterone versus testosterone only [2 groups]<br><br>PRE+TESTOSTERONE versus TESTOSTERONE ONLY	33	0%	80%	30	3/33 (9%)	PRE NTERVENTION: Upper and lower body training using free weights. PRE included warm-up, 5-8 reps at 50% 1-RM for each exercise; 3 sets of 8 reps at 80% 1-RM with the final set performed to failure, with 2 min rest in between all sets. Participants started at 70% 1-RM at baseline and increased to 80% 1-RM by the end of the 2nd week. 1-RM was assessed every 2 weeks to adjust training load to maintain intensity at 80% 1-RM.	3X per week for 12 weeks	Exercise lab	Supervised

Additional File 2: Detailed Characteristics of Included Studies in the Progressive Resistive Exercise (PRE) and HIV Systematic Review (n=20 studies)

Study	Methods	Sample Size (at baseline)	% Women	% taking combination ART	Participants (at study completion)	Withdrawal Rate	Intervention	Duration and Frequency	Location of Exercise	Supervision
Sattler (1999) (continued)	PRE+TESTOSTERONE versus TESTOSTERONE ONLY						TESTOSTERONE INTERVENTION: Weekly injections of nandrolone; 200mg in week 1; 400mg in week 2; 600 mg for weeks 3-12.			
Shevitz (2005)	Randomized combined nutrition plus exercise, nutrition plus oxandrolone, and nutrition only [3 groups]<br><br>PRE + NUTRITION + OXANDROLONE VERSUS NUTRITION + OXANDROLONE ONLY VERSUS NUTRITION ONLY	50 (with wasting)	30%	80%	47	3/50 (6%)	PRE INTERVENTION GROUP: Resistance training of six major muscle groups of upper and lower body (leg press, chest press, knee extension, seated row, leg press): 3 sets of 8 repetitions each with progressive increase in intensity to 80% 1-RM on each (modified abdominal curl-ups): 2 sets of 10 repetitions each with gradual increase with weight plate over chest. Strength was assessed at sessions 3 and 25 to adjust resistive intensity accordingly.	3 x/week frequency for 12 weeks	*NR*	Supervised

Additional File 2: Detailed Characteristics of Included Studies in the Progressive Resistive Exercise (PRE) and HIV Systematic Review (n=20 studies)

Study	Methods	Sample Size (at baseline)	% Women	% taking combination ART	Participants (at study completion)	Withdrawal Rate	Intervention	Duration and Frequency	Location of Exercise	Supervision
Shevitz (2005) (continued)	PRE + NUTRITION + OXANDROLONE VERSUS NUTRITION + OXANDROLONE ONLY VERSUS NUTRITION ONLY						OXANDROLONE: 10mg tablets taken twice daily.<br><br>NUTRITION: Weekly visits 30-60 minutes in duration. Encouraged to meet dietary recommendations based on weight to promote weight gain; 40-50 kcal/kg/day calories and 1.6g/kg/day protein, oral liquid supplement 2 times daily that contained 240 kcal, 15g of protein and 3g of fat. Weekly visits counselled on good dietary intake.			

Additional File 2: Detailed Characteristics of Included Studies in the Progressive Resistive Exercise (PRE) and HIV Systematic Review (n=20 studies)

Study	Methods	Sample Size (at baseline)	% Women	% taking combination ART	Participants (at study completion)	Withdrawal Rate	Intervention	Duration and Frequency	Location of Exercise	Supervision
Spence (1990)	Randomized exercise and control groups [2 groups]<br><br>PRE VERSUS NON-EXERCISING CONTROL	24	0%	100% taking AZT	NR	Unknown	PRE INTERVENTION GROUP: Resistance training (bilateral and bi-directional concentric contractions throughout range of motion). *Progression of PRE Intensity*: Resistance loading was uniformly increased throughout the training period from 1 set of 15 repetitions on the minimum setting of the Total Power hydraulic resistance training unit, through to 3 sets of 10 repetitions on the maximum setting. Maximum effort was encouraged.<br><br>NON-EXERCISING CONTROL GROUP: Usual care.	Duration unknown; 3 x/week for 6 weeks	NR	Supervised

Additional File 2: Detailed Characteristics of Included Studies in the Progressive Resistive Exercise (PRE) and HIV Systematic Review (n=20 studies)

Study	Methods	Sample Size (at baseline)	% Women	% taking combination ART	Participants (at study completion)	Withdrawal Rate	Intervention	Duration and Frequency	Location of Exercise	Supervision
Tiozzo (2011)* #	Randomized exercise and control groups [2 groups]<br><br>CONSTANT AEROBIC + PRE VERSUS NON-EXERCISING CONTROL	37	39%	100%	23	14/37 (38%)	EXERCISE (AEROBIC + PRE) INTERVENTION GROUP: Moderate Intensity<br>*Aerobic Exercise*: Week 1 and 2: These 2 weeks were a phase-in period allowing participants to acclimate to the exercise protocol. This consisted of 3 endurance sessions, 5 minute warm up and cool down periods and 10-15 min of aerobic exercise on a stationary treadmill or bike ergometer at an intensity of 60% maximal HR.<br>*Progression of Aerobic Intensity:* After the initial 60% of aerobic training intensity and 60% of 1RM resistance training intensity during the phase in period, intensity was gradually increased to 65% of	3X per week for 12 weeks	Wellness medical centre	Supervised

Additional File 2: Detailed Characteristics of Included Studies in the Progressive Resistive Exercise (PRE) and HIV Systematic Review (n=20 studies)

Study	Methods	Sample Size (at baseline)	% Women	% taking combination ART	Participants (at study completion)	Withdrawal Rate	Intervention	Duration and Frequency	Location of Exercise	Supervision
Tiozzo (2011)* # (continued)	CONSTANT AEROBIC + PRE VERSUS NON-EXERCISING CONTROL						HRmax and 65% of 1RM in Step 1, to 70% in Step 2 and to 75% in Step 3.<br><br>*PRE*: All endurance sessions followed immediately by core consisting of 8 two to three sets of 15 to 20 reps, and one set of 12 reps for ten exercises performed on stacked weight machines. The initial level for the resistance exercises was 60% of one repetition maximum (1RM). *Progression of PRE Intensity*: Step 1 consisted of high reps (12), followed by lower reps in Step 2 and Step 3 (10 and 8 reps, respectively). Similar to the phase-in period, other phases allocated the same amount of time to each component (aerobic versus			

Additional File 2: Detailed Characteristics of Included Studies in the Progressive Resistive Exercise (PRE) and HIV Systematic Review (n=20 studies)

Study	Methods	Sample Size (at baseline)	% Women	% taking combination ART	Participants (at study completion)	Withdrawal Rate	Intervention	Duration and Frequency	Location of Exercise	Supervision
Tiozzo (2011)* # (continued)	CONSTANT AEROBIC + PRE VERSUS NON-EXERCISING CONTROL						resistance) of the exercise program. <u>NON-EXERCISING CONTROL GROUP:</u> Participants were asked not to participate in any form of exercise.			
Yarasheski (2011)* #	Randomized exercise + pioglitazone versus pioglitazone only [2 groups]<br><br>CONSTANT AEROBIC + PRE + PIOGLITAZONE VERSUS PIOGLITAZONE ONLY	44 (with insulin resistance, impaired glucose intolerance and central adiposity)	13%	100%	39	5/44 (11%)	<u>EXERCISE (AEROBIC + PRE) PLUS PIOGLITAZONE GROUP:</u> *Aerobic Exercise*: Stationary cycling, treadmill walk/jogging, stair stepper climbing, or elliptical training device. Target HR range during aerobic exercise was 50-85% HR reserve (moderate to high intensity). During exercise, HR and time at the target HR were monitored. Signaled an alarm if target HR was not maintained.HR and time data were stored to verify adherence and response to the exercise. Trainer	1.5 to 2 hours per session; 3X per week for 16 weeks	Indoor exercise facility	Supervised

Additional File 2: Detailed Characteristics of Included Studies in the Progressive Resistive Exercise (PRE) and HIV Systematic Review (n=20 studies)

Study	Methods	Sample Size (at baseline)	% Women	% taking combination ART	Participants (at study completion)	Withdrawal Rate	Intervention	Duration and Frequency	Location of Exercise	Supervision
Yarasheski (2011)* # (continued)	CONSTANT AEROBIC + PRE + PIOGLITAZONE VERSUS PIOGLITAZONE ONLY						progressively increased the exercise intensity as the participants adapted.<br><br>*PRE*: 4 upper and 3 lower body exercises following the aerobic session. Baseline 1 repetition maximum was measured during the 1st 3-4 exercise sessions on each of the machines. Initially PRE consisted of 1-2 sets of each exercise while lifting a weight that caused muscle fatigue / failure after 8 repetitions. The trainer monitored the participant's exercise response daily and when the participant comfortably lifted the weight for 12 reps on any exercise, the weight (intensity)			

Additional File 2: Detailed Characteristics of Included Studies in the Progressive Resistive Exercise (PRE) and HIV Systematic Review (n=20 studies)

Study	Methods	Sample Size (at baseline)	% Women	% taking combination ART	Participants (at study completion)	Withdrawal Rate	Intervention	Duration and Frequency	Location of Exercise	Supervision
Yarasheski (2011)* # (continued)	CONSTANT AEROBIC + PRE + PIOGLITAZONE VERSUS PIOGLITAZONE ONLY						was increased by an amount ~10% that caused the muscle group to fatigue / fail after 8 reps. This progressive 8-12 repetition cycle was repeated for each exercise over the 4 month period.<br><br><u>PIOGLITAZONE ONLY GROUP</u>: Participants consumed a standard weight diet that contained adequate amounts of energy and macronutrients.			

*study included in this recent update of the systematic review;

#study included in systematic review examining effect of aerobic exercise with adults living with HIV [12] https://bmcinfectdis.biomedcentral.com/articles/10.1186/s12879-016-1478-2;

~For the purpose of this review, only the PRE and control groups were included in meta-analyses;

PRE=progressive resistive exercise; AER=aerobic exercise; NR=not reported; ART=antiretroviral therapy; cART=combination antiretroviral therapy; HAART=highly active antiretroviral therapy; 1RM=1 repetition maximum; HR= heart rate; reps=repetitions.
