# Supplementary material for: Effectiveness of Progressive Resistive Exercise (PRE) in the context of HIV: systematic review and meta-analysis using the Cochrane Collaboration protocol
Source: BMC Infect Dis. 2017 Apr 12;17:268. doi: 10.1186/s12879-017-2342-8 (PMC5389006; doi:10.1186/s12879-017-2342-8)
Supplement: Supplementary file 3 — Details of Outcomes and Authors’ Conclusions of Individual Studies Included in the Progressive Resistive Exercise (PRE) and HIV Systematic Review (n = 20 studies) (PDF 205 kb) [file 12879_2017_2342_MOESM3_ESM.pdf]

Additional File 3: Details of Outcomes and Authors' Conclusions of Individual Studies Included in the Progressive Resistive Exercise (PRE) and HIV Systematic Review (n=20 studies)

Study	Immunological and Virological	Cardiorespiratory	Strength	Weight and Body Composition	Psychological	Adverse Events	Authors' Conclusions
Agin (2001)	Not assessed	Not assessed	*Upper and lower extremity strength*: Increase in muscle strength ranging from 41-95% across all 7 muscle groups assessed in the PRE only and combined PRE and whey protein groups.	*Weight:* Significant increase in body weight in the whey protein group.<br><br>*Body Composition:* Significant increase in body cell mass in both the PRE only and combined PRE and whey protein groups. *Fat Mass:* Significant increase in the whey only group and significantly decreased in the PRE only group, with no change in the combined PRE and whey protein group. *Fat Free Mass:* Significant increase in all three groups.	*Health-related quality of life:* (as measured by the MOS-HIV Scale) Significant increase in the physical activity scores for the PRE only group and significant decrease in the whey only and combined PRE and whey groups. Significant improvement in the PRE group for general health perception and vitality sub-scale scores.	One death was reported in the combined PRE and whey group. No injuries were reported from PRE or the assessments.	Resistance exercise significantly increased body cell mass, muscle mass, muscle strength and HRQL in women living with HIV and reduced body cell mass. Whey protein had little effect on body cell mass gain and combined PRE and whey protein did not increase body cell mass beyond gains achieved by PRE alone.
Agostini (2009)* #	Not assessed	Not assessed	Not assessed	*Weight:* Assessed but data not available.<br><br>*Body Fat*: Decrease in abdominal fat was similar in both groups. There does not appear to be a significant difference between groups.	Not assessed	Not reported	Aerobic exercise and a balanced diet are key pillars in the non-pharmacological treatment of lipodystrophy.

Additional File 3: Details of Outcomes and Authors' Conclusions of Individual Studies Included in the Progressive Resistive Exercise (PRE) and HIV Systematic Review (n=20 studies)

Study	Immunological and Virological	Cardiorespiratory	Strength	Weight and Body Composition	Psychological	Adverse Events	Authors' Conclusions
Balasubramanyam (2011)* #	*CD4 count and viral load*: No significant differences between groups.	No significant difference between groups for VCO2, VO2, respiratory quotient, resting energy expenditure.	Not assessed	*Weight*: No statistically significant difference between groups.<br><br>*Body composition*: No significant difference between groups for body mass index (kg/m2), waist circumference, hip circumference, waist to hip ratio, body cell mass, fat mass (kg) and body fat (%). As intended in the Diet and Exercise (weight maintaining lifestyle intervention), there were no significant changes within groups or between groups in weight or BMI.	Not assessed	Adverse events were reported in both groups. Exercise Group: 24 adverse events reported in at least 1% of participants ranging from (but not limited to) events such as diarrhea, nausea and vomiting, fatigue, dizziness, and headache. Recommendation Group: 20 adverse events reported in at least 1% of participants ranging from (but not limited to) events such as: triglyceride >1000 mg/dl, elevated bilirubin, abdominal pain.	The combination of niacin and fenofibrate together with diet and exercise (D/E) is more effective than lifestyle change alone or drug monotherapy with lifestyle change in improving HIV associated dyslipidemia. Diet and Exercise intervention alone did not improve lipid levels or adiponectin or induce statistically significant changes in any of the secondary (body composition) outcomes.

Additional File 3: Details of Outcomes and Authors' Conclusions of Individual Studies Included in the Progressive Resistive Exercise (PRE) and HIV Systematic Review (n=20 studies)

Study	Immunological and Virological	Cardiorespiratory	Strength	Weight and Body Composition	Psychological	Adverse Events	Authors' Conclusions
Bhasin (2000)	*CD4 count and viral load*: No significant changes.	Not assessed	*Upper and lower extremity strength*: Increases in the PRE only group by 29-36%, and increases in the testosterone only group by 17-28% for 5 outcomes of strength. Increases in the combined PRE and testosterone group by 10-32% (which were non-significantly greater than either intervention alone). No changes in the non-exercising control group.	*Weight:* Significant increase by 2.6kg in men receiving testosterone alone; increase by 2.2kg in men who exercised alone; no changes in the control group. Combined PRE and testosterone group did not show a greater increase in weight compared to one intervention alone. *Body Composition: Fat Free Mass:* Significant increase in the testosterone and PRE only groups. No change in the non-exercising control group. Combined PRE and testosterone group did not show a greater increase in weight compared to one intervention alone. *Muscle Area*: Greater increase in thigh muscle area in testosterone only (40cm^3), PRE only (62cm^3), combined testosterone and PRE group (44cm^3) (compared with placebo (5cm^3). *Lean Body Mass*:Increased in the testosterone only (2.3kg), and combined PRE and testosterone groups (2.6kg); no change in the non-exercising control.	*Health-related quality of life:* No association between the change in HRQL scores and testosterone administration or exercise administration were found in any of the groups.	No changes in immunological, physiological, or virological outcomes. One participant in the testosterone group and one participant in the placebo group developed acne. One participant in the testosterone group developed breast enlargement. No withdrawals were attributed to adverse events.	Testosterone and resistance exercise promotes gains in body weight, muscle mass, muscle strength and lean body mass among men living with HIV with moderate weight loss and low testosterone levels. The effect of testosterone and exercise were not additive in this study.

Additional File 3: Details of Outcomes and Authors' Conclusions of Individual Studies Included in the Progressive Resistive Exercise (PRE) and HIV Systematic Review (n=20 studies)

Study	Immunological and Virological	Cardiorespiratory	Strength	Weight and Body Composition	Psychological	Adverse Events	Authors' Conclusions
Dolan (2006) #	*CD4 count and viral load*: No significant changes.	*6MWT*: Significant improvements in exercise time as measured by submaximal exercise time and 6MWT distance among exercisers compared with non-exercisers.<br><br>*VO2max*: Significant improvements among exercisers compared with non-exercisers.	*Upper and lower extremity strength*: Significant improvements in upper and lower extremity strength (7 measures) among exercisers compared with non-exercisers.	*Weight*: No significant change between groups.<br><br>*Body Composition:* Significant increase in total cross-sectional muscle area and muscle attenuation among exercisers compared with non-exercisers. Significant decrease in waist circumference among exercisers compared with non-exercisers. No significant difference between group for body mass index, abdominal visceral tissue area, subcutaneous adipose tissue area, and total fat.	Not assessed	Authors reported 1 participant who had an exacerbation of asthma, and 1 participant had chest pain but neither were related to exercise.	A 16 week supervised home based PRE and aerobic exercise program improves measures of strength, cardiorespiratory fitness, and body composition among women living with HIV.
Driscoll (2004a) #	*CD4 count and viral load*: No significant changes.	*Exercise Time*: Significant improvements in endurance time on cycle ergometer during submaximal stress test in the exercise and metformin group compared with the metformin only group.	Significant increases in upper and lower extremity strength (five of six indices) in the exercise and metformin group compared with the metformin only group.	*Weight*: No significant changes in either group.<br><br>*Body Composition*: Significant increases in cross-sectional muscle area, and significant decreases in waist-to-hip ratio and abdominal fat area in the exercise and metformin group compared with the metformin only group. No significant changes in body mass index in either group.	Not assessed	None reported	Exercise training and metformin improved cardiovascular outcomes more than metformin alone in persons living with HIV with fat redistribution and hyperinsulinemia. Exercise training (aerobic and PRE) is well-tolerated and improves muscle strength and size as well as aerobic fitness in persons living with HIV.

Additional File 3: Details of Outcomes and Authors' Conclusions of Individual Studies Included in the Progressive Resistive Exercise (PRE) and HIV Systematic Review (n=20 studies)

Study	Immunological and Virological	Cardiorespiratory	Strength	Weight and Body Composition	Psychological	Adverse Events	Authors' Conclusions
Farinatti (2010)* #	*CD4 count*: No significant changes in Cd4 count or CD4% within or between groups.	Significant improvements within exercisers and significantly greater improvements among exercisers compared with non-exercisers (slope and intercept for HR-workload).	Significant improvement within exercisers and significantly greater improvements in leg press (12-RM) and seated row (12-RM) among exercisers compared with non-exercisers.	*Weight*: Not assessed<br><br>*Body Composition*: No significant difference within or between groups for body mass index (kg/mg) or body mass (kg).	Not assessed	None reported	HIV infected patients treated with HAART improve their strength and aerobic fitness as a result of a supervised exercise program of aerobic, strength and flexibility exercises with no negative effect on immune function.
Fitch (2012)* #	*CD4 count and viral load*: No significant differences between groups.	*VO2max and Endurance Time*: Improvements in exercisers compared with non exercisers. No significant effect of metformin on cardiopulmonary measures. Significantly greater improvement in VO2max among the combined Metformin+Exercise group versus the control group. ($p=0.05$). Significantly greater improvement in exercise duration (min) among the exercising groups (EXERCISE only group) and	Exercise was associated with improvements in all strength parameters ($p<0.01$) compared with non-exercisers. Significantly greater improvement in triceps strength, knee flexor strength, lat pull down, knee extension strength, chest press, leg press, among the exercising groups (EXERCISE only group) and (EXERCISE + METFORMIN group) versus	*Weight*: Not assessed<br><br>*Body Composition*: Intramyocellular lipid (IMCL) improved in exercisers compared to non-exercisers. Visceral adipose tissue decreased in participants randomized to metformin only versus control, although this was not significant. Extremity fat did not change significantly in response to exercise or metformin.<br><br>Significant between group difference between the exercise and control groups ($p<0.05$) whereby the exercise group had greater reduction in tibialis anterior intramyocellular lipid (IMCL) compared with	Not assessed	Two participants in the exercise group experienced muscle strains related to the resistance training necessitating modification of weights. There were no serious adverse events and the exercise program was well-tolerated.	Metformin participants demonstrated significantly less progression of coronary artery calcification (CAC) whereas the effect of exercise on CAC progression was not significant. Metformin had a significantly greater effect on CAC than exercise.<br><br>Exercise participants showed significant improvement in HDL, and cardiorespiratory fitness compared to non-exercisers. Metformin prevents plaque progression

Additional File 3: Details of Outcomes and Authors' Conclusions of Individual Studies Included in the Progressive Resistive Exercise (PRE) and HIV Systematic Review (n=20 studies)

Study	Immunological and Virological	Cardiorespiratory	Strength	Weight and Body Composition	Psychological	Adverse Events	Authors' Conclusions
Fitch (2012)* # (continued)		(EXERCISE + METFORMIN group) versus control. Significantly greater increase in exercise duration among the EXERCISE only group versus METFORMIN only group. (p=0.006).	control. Significantly greater increase in triceps strength, knee flexor strength, lat pull down, knee extension strength, chest press, leg press, among the EXERCISE only group versus METFORMIN only group.	control. Significant difference between the exercise and metformin only group whereby the exercise group had a greater reduction in tibialis anterior IMCL compared with the metformin only group. Assuming that reduction in cellular lipid is a good outcome this suggests exercise had a beneficial effect beyond control and metformin only for reducing cellular lipid. No significant difference between groups for change in body mass index (kg/m2), visceral adipose tissue (cm2), subcutaneous adipose tissue (cm2), total extremity fat (kg), and waist circumference (cm).			in HIV infected individuals with metabolic syndrome. Exercise demonstrates improvements in cardiopulmonary fitness and strength.

Additional File 3: Details of Outcomes and Authors' Conclusions of Individual Studies Included in the Progressive Resistive Exercise (PRE) and HIV Systematic Review (n=20 studies)

Study	Immunological and Virological	Cardiorespiratory	Strength	Weight and Body Composition	Psychological	Adverse Events	Authors' Conclusions
Grinspoon (2000) #	*CD4 count and viral load*: No significant changes with exercise or testosterone therapy either alone or together as a co-intervention.	Not assessed	No significant change in strength (note strength was tested isometrically, which may underestimate change in strength).	*Weight:* No significant changes in either group.<br><br>*Body Composition:* Participants in the exercise only group showed significant increases in lean body mass, arm muscle area, leg muscle area, HDL cholesterol and significant decreases in AST level compared to non-exercising control group. No significant changes in and fat mass in either the exercisers or non-exercising control group.	Not assessed	No deaths or adverse events.	Exercise has a significant effect on lean body mass and muscle area independent of testosterone. Muscle mass and strength may further increase in response to combined exercise and testosterone therapy. Exercise was associated with an increase in HDL cholesterol whereas testosterone decreased HDL cholesterol. Exercise significantly increases muscle mass and offers cardio protective effects by increasing the HDL cholesterol in men with AIDS wasting. Exercise may be a strategy to reverse muscle loss in this population.

Additional File 3: Details of Outcomes and Authors' Conclusions of Individual Studies Included in the Progressive Resistive Exercise (PRE) and HIV Systematic Review (n=20 studies)

Study	Immunological and Virological	Cardiorespiratory	Strength	Weight and Body Composition	Psychological	Adverse Events	Authors' Conclusions
Lindegaard (2008)* #	Not reported	*VO2max:* Significant increase in VO2max by 14.4% in the aerobic group with no difference in the PRE group. Greater improvement in VO2max in the AEROBIC group versus the PRE group.	Significant increase in strength by 30% in the PRE group and by 7.8% in the aerobic group. The increase was more pronounced after strength training than after aerobic training.	*Weight:* PRE group had significant decrease in body weight whereas the aerobic group demonstrated no change.<br><br>*Body Composition*: PRE group had a significant increase in lean body mass, decreased total fat and limb fat mass whereas the aerobic group demonstrated no changes in these outcomes.	Not assessed	Not reported	Strength training and endurance training improved insulin mediated glucose uptake but only in the PRE group and not AEROBIC group and caused a reduction in total fat mass. In conclusion, both AEROBIC and PRE training increases insulin sensitivity in HIV-infected patients with lipodystrophy whereas only strength training reduces trunk fat mass. Authors suggest an appropriate exercise program should include PRE and AER training to reduce the risk of cardiovascular disease among people with lipodystrophy.

Additional File 3: Details of Outcomes and Authors' Conclusions of Individual Studies Included in the Progressive Resistive Exercise (PRE) and HIV Systematic Review (n=20 studies)

Study	Immunological and Virological	Cardiorespiratory	Strength	Weight and Body Composition	Psychological	Adverse Events	Authors' Conclusions
Lox (1995) #	*CD4 count and viral load*: No significant changes.	*VO2max:* Significant improvements among exercisers compared to non-exercisers with greater improvements in the aerobic compared to the PRE and non-exercising control groups.<br><br>*Heart Rate:* Non-significant decrease in submaximum HR in the PRE group compared to a non-significant increase in the non-exercising control group.	Significant improvements in the PRE and aerobic exercise groups compared to the non-exercising control groups. Significantly greater improvements as measured by 1-RM in the PRE group compared to the aerobic and non-exercising control groups.	*Weight*: Significant increases in weight among PRE and aerobic exercise groups.<br><br>*Body Composition*: No change among all 3 groups in average body mass index, fat mass, and body fat percentage. Significant increases in lean body mass and sum of chest, arm and thigh circumference among PRE and aerobic exercise groups.	*Mood and Life Satisfaction:* Significant improvements in mood and life satisfaction in both the aerobic and PRE exercise groups compared to the non-exercising control group. Significantly higher life satisfaction in the aerobic group compared with the PRE group.	Not reported	Exercise results in improvements in body composition, strength, cardiopulmonary fitness, and mood and life satisfaction for people living with HIV.

Additional File 3: Details of Outcomes and Authors' Conclusions of Individual Studies Included in the Progressive Resistive Exercise (PRE) and HIV Systematic Review (n=20 studies)

Study	Immunological and Virological	Cardiorespiratory	Strength	Weight and Body Composition	Psychological	Adverse Events	Authors' Conclusions
Ogalha (2011)* #	*CD4 count:* Significant improvement in CD4 count in both groups.	*VO2max:* 'Marginally' significant (p=0.05) improvement in VO2max for exercisers only.<br><br>Statistically significant improvement (reduction) in resting heart rate in the exercise group only (within group difference).	Not assessed	*Weight:* No significant within or between group differences for body weight.<br><br>*Body Composition:* Statistically significant improvement in muscle mass, body fat percent, hip circumference (decrease) among the exercisers (within group difference only). Statistically significant improvement in BMI, and hip circumference (decrease) among the control group (within group difference). No significant difference within or between groups for waist circumference or waist to hip ratio.	*Quality of Life:* All SF36 domain scores improved significantly similarly for all domains in both groups except for the pain domain (whereby the control group was the only group to show significant improvement). Improvements in QOL were significantly greater for the exercise group compared with the control group for general health, vitality, and mental health.	Not reported	Regular exercise coupled with nutritional guidance in people living with HIV significantly improves quality of life. Main findings suggest that the intervention promoted significant modifications in increase in muscle mass and reduction in fasting glucose, BMI, body fat, and hip circumference.

Additional File 3: Details of Outcomes and Authors' Conclusions of Individual Studies Included in the Progressive Resistive Exercise (PRE) and HIV Systematic Review (n=20 studies)

Study	Immunological and Virological	Cardiorespiratory	Strength	Weight and Body Composition	Psychological	Adverse Events	Authors' Conclusions
Perez-Moreno (2007)* #	*CD4 count:* Significant increase in CD4 count among exercisers (within group only).	Statistically significant improvement in peak workload (Watts) among exercisers whereas there was a significant decrease (worsening) in the control group.<br><br>*HRmax:* Significant improvement in heart rate peak (bpm) among exercisers. A significant combined effect of group and time was found for peak-completed workload (W), HRpeak, and rate of HR decrease at 1-min post exercise compared to attained HRpeak among exercisers.	Significant improvement among exercisers for strength whereas no change among non-exercisers. Significant improvement in the upper and lower body dynamic strength endurance (6RM) among exercisers (bench press, knee extensor strength) compared with non-exercisers.	*Weight*: Not assessed<br><br>*Body Composition*: No significant changes within groups for body mass. Mean estimated muscle mass significantly increased in the exercise group (within group only) with no change in the control group.	*Quality of Life:* Statistically significant improvement in QOL as measured by the QOL Assessment with a Scale from Spain in the exercise group ($p<0.01$) whereas no change occurred in the control group.	No major adverse effects and no major health problems were noted in the participants from both groups over the training period.	A combination of cardiorespiratory and resistance training produces significant gains in cardiorespiratory capacity and dynamic strength endurance of incarcerated men who are HIV-HepC co-infected and enrolled in a methadone maintenance program for the treatment of opioid dependency.

Additional File 3: Details of Outcomes and Authors' Conclusions of Individual Studies Included in the Progressive Resistive Exercise (PRE) and HIV Systematic Review (n=20 studies)

Study	Immunological and Virological	Cardiorespiratory	Strength	Weight and Body Composition	Psychological	Adverse Events	Authors' Conclusions
Rigsby (1992)	*CD4 count:* No significant changes.	*Aerobic Capacity:* Significant increases in aerobic capacity were shown in the exercise group with no change in non-exercising control group.<br><br>*Heart Rate and Total Time to Voluntary Exhaustion:* Significant decreases in HR and increases in total time exercise to voluntary exhaustion.	Significant increases in chest press and leg extension in the exercise group.	Not assessed	Not assessed	One death reported in the counselling group during the course of the study and one death one month after the study. Of the 4 participants who dropped out of the exercise group, one died immediately after the study conclusion.	HIV+ men can experience significant increases in neuromuscular strength and cardiorespiratory fitness when prescribed and monitored in accordance with ACSM guidelines for healthy adults. Increased fitness may occur without negative effects on immune status.

Additional File 3: Details of Outcomes and Authors' Conclusions of Individual Studies Included in the Progressive Resistive Exercise (PRE) and HIV Systematic Review (n=20 studies)

Study	Immunological and Virological	Cardiorespiratory	Strength	Weight and Body Composition	Psychological	Adverse Events	Authors' Conclusions
Sakkas (2009)*	Not assessed	*Fatigue:* Fatigue profile (defined as the rate of force decline during the 6 minute exercise protocol) did not differ between or within the 2 groups at any time point.	*Upper and Lower Body Strength*: Significant increase in strength within both the PRE+creatine (44%) and PRE only (42%) groups for upper and lower body strength. No significant difference between groups.	*Weight*: Increase in the creatine group only.<br><br>*Body Composition:* Significant increase in lean body mass in both groups but a significantly greater between group increase in lean body mass in the PRE+creatine group compared with the PRE only group. Significant increase in limb (arm and leg), lean body mass, and thigh muscle cross sectional area within both groups.<br><br>*Fat*: No significant change within groups for total body fat, trunk fat, arm and leg fat, extramyocellular fat, thigh subcutaneous fat, cell muscle cross sectional area, calf subcutaneous adipose tissue area. No differences between groups for total, trunk or limb fat.	Not assessed	Not reported	PRE consistently increased muscle strength among men living with HIV. Although participants who received PRE as well as CREATINE supplementation had a greater increase in lean body mass, results provide no evidence that creatine augmented the increase in strength derived from PRE. The use of creatine supplementation in this sample may be limited to aesthetic purposes rather than to improve functional capacity. The efficacy and safety of PRE demonstrates its potential benefit in preventing or reversing muscle weakness.

Additional File 3: Details of Outcomes and Authors' Conclusions of Individual Studies Included in the Progressive Resistive Exercise (PRE) and HIV Systematic Review (n=20 studies)

Study	Immunological and Virological	Cardiorespiratory	Strength	Weight and Body Composition	Psychological	Adverse Events	Authors' Conclusions
Sattler (1999)	*CD4 count:* Non-significant increases for both intervention groups.	Not assessed	*Upper and Lower Extremity Strength:* Significant increases in upper and lower body strength in both groups with significantly greater increases in strength in the combined PRE and testosterone group (14-53%) compared with the testosterone only group (10-31%).	*Weight:* Significant increases in both groups (PRE and combined PRE+ testosterone).<br><br>*Body Composition:* Significant increases in thigh muscle area and body cell mass within both groups; no difference between groups. Significant decrease in fat mass in the combined PRE+ testosterone group (no change in the testosterone only group). Significantly greater increases in lean body mass in the combined PRE+ testosterone group.	Not assessed	Acne and testicular shrinkage were found in testosterone groups.	Testosterone resulted in significant increases in total weight, lean body mass, body cell mass, muscle size and strength. Increases in lean body mass and muscular strength were significantly greater with PRE.

Additional File 3: Details of Outcomes and Authors' Conclusions of Individual Studies Included in the Progressive Resistive Exercise (PRE) and HIV Systematic Review (n=20 studies)

Study	Immunological and Virological	Cardiorespiratory	Strength	Weight and Body Composition	Psychological	Adverse Events	Authors' Conclusions
Shevitz (2005)	*CD4 count and viral load:* No significant changes.	*Endurance Tolerance:* Significant improvements in endurance tolerance in all 3 groups but significantly greater improvements in six minute walk test distance and chair stand time in the combined PRE+ nutrition group versus nutrition only group. Significant improvements in self-reported function in the combined PRE+ nutrition group only.	*Upper and Lower Extremity Strength:* Significant improvements in all 7 upper and lower extremity strength measures in the combined PRE+ nutrition group, 1/7 strength measures for the nutrition only group, and 2/7 measures for the combined oxandrolone+ nutrition group.	*Weight:* Significant increase within all three groups. No significant difference between groups.<br><br>*Body Composition:* No changes in body mass index across all 3 groups. Fat Free Mass: Significant increase in fat free mass within the combined oxandrolone+ nutrition group and the nutrition only group. Significant increase in mid-thigh cross-sectional muscle area within the combined PRE+ nutrition group and the combined oxandrolone + nutrition group. No significant differences between groups for body composition.	*Quality of Life Adjusted Years*: No significant change in Quality of Life Adjusted Years within groups but the increase was greatest in the PRE+ nutrition group demonstrating most favourable cost-effectiveness.	None reported	Oxandrolone and PRE demonstrate similar improvements in body composition however PRE is superior over oxandrolone to improve strength, physical function, lean body mass and dietary intake with a lower cost and low risk of adverse effects.

Additional File 3: Details of Outcomes and Authors' Conclusions of Individual Studies Included in the Progressive Resistive Exercise (PRE) and HIV Systematic Review (n=20 studies)

Study	Immunological and Virological	Cardiorespiratory	Strength	Weight and Body Composition	Psychological	Adverse Events	Authors' Conclusions
Spence (1990)	Not assessed	Not assessed	*Upper and Lower Extremity Strength:* Significant increases in all lower extremity (12/12) and most upper extremity variables (10/12) in the exercise group. Significant decreases in upper extremity (6/12) and lower extremity (7/12) variables in the non-exercising control group.	*Weight*: Significant increases in weight compared with the non-exercising control group (which showed decreases in weight).<br><br>*Body Composition:* Significant increases in arm and thigh girth compared with the control group which showed decreases in girth. No differences for sum of skinfolds.	Not assessed	Not reported	PRE improved muscle function and anthropometry in persons living with HIV in the PRE group compared to persons living with HIV in the non-exercising control group. *Note weight gain was considered a favourable outcome in this study*

Additional File 3: Details of Outcomes and Authors' Conclusions of Individual Studies Included in the Progressive Resistive Exercise (PRE) and HIV Systematic Review (n=20 studies)

Study	Immunological and Virological	Cardiorespiratory	Strength	Weight and Body Composition	Psychological	Adverse Events	Authors' Conclusions
Tiozzo (2011)* #	*CD4 count and Viral Load:* Significant decrease in CD4 count among non-exercisers (control group) whereas CD4 count remained the same in the exercise group. Exercisers had significantly greater CD4 count at study completion compared with non-exercisers. No significant changes in viral load in either group.	*VO2max:* Significant increase (improvement) in VO2max compared with non-exercisers.<br><br>*HRmax*: No difference in heart rate or diastolic blood pressure within or between groups. Significant difference between groups at baseline for systolic blood pressure - the exercise group had lower systolic blood pressure at baseline but at study completion the control group had lowered their systolic blood pressure.	Significant difference within exercisers who demonstrated an increase in 1RM chest and 1 RM legs whereas there was no change in the control group. Significantly greater improvement in 1RM chest among exercisers compared with control.	*Weight:* No significant changes.<br><br>*Body Composition:* No significant changes in hip circumference or waist-to-hip ratio in either the exercise or control group. Significant reduction in waist circumference among exercisers whereas the non-exercisers waist circumference increased.	*Quality of Life:* Exercisers had significant improvements in SF36 physical function sub scale and mental health sub scale, compared with non-exercisers who demonstrated a significant worsening from baseline.	Not reported	A three month supervised, and moderate intensity cardiorespiratory and resistance exercise training program performed three times a week, can result in significant improvements in physical characteristics and physical fitness and QOL among people living with HIV.

Additional File 3: Details of Outcomes and Authors' Conclusions of Individual Studies Included in the Progressive Resistive Exercise (PRE) and HIV Systematic Review (n=20 studies)

Study	Immunological and Virological	Cardiorespiratory	Strength	Weight and Body Composition	Psychological	Adverse Events	Authors' Conclusions
Yarasheski (2011)* #	*CD4 count and Viral Load:* No significant changes.	Not assessed	Not assessed	*Weight:* No significant change in body weight within groups.<br><br>*Body Composition:* Significant increase in thigh muscle area among exercisers compared with non-exercisers (within and between group difference) and non-exercisers had a decrease in thigh muscle area.<br><br>No other significant within or between group differences in other body composition outcomes: body mass index, fat mass, fat free mass, trunk fat mass, limb fat mass, visceral adipose tissue, abdominal adipose tissue, right and left thigh subcutaneous fat, total hip bone mineral density, lumbar spine bone mineral density, hip or lumbar z-score.	Not assessed	No serious adverse events or complications reported	Overall, combined exercise intervention for diabetes prevention that includes diet and exercise is more effective than medication interventions alone.

*study included in this recent update of the systematic review

#study included in systematic review examining effect of aerobic exercise with adults living with HIV [12] https://bmcinfectdis.biomedcentral.com/articles/10.1186/s12879-016-1478-2;

HRQL=health-related quality of life; MOS-HIV=Medical Outcomes Study HIV Scale; VO2max=maximum oxygen consumption; 6MWT=6 minute walk test; BMI=body mass index; 1-RM=1 repetition maximum;
