# Supplementary material for: Effectiveness of Progressive Resistive Exercise (PRE) in the context of HIV: systematic review and meta-analysis using the Cochrane Collaboration protocol
Source: BMC Infect Dis. 2017 Apr 12;17:268. doi: 10.1186/s12879-017-2342-8 (PMC5389006; doi:10.1186/s12879-017-2342-8)
Supplement: Supplementary file 4 — GRADE Summary of Finding Table: Progressive Resistive Exercise (PRE) or Combined Progressive Resistive Exercise (PRE) and Aerobic Exercise Compared with No Exercise for adults living with HIV (PDF 111 kb) [file 12879_2017_2342_MOESM4_ESM.pdf]

Additional File 4 - GRADE Summary of Finding Table: Progressive Resistive Exercise (PRE) or Combined Progressive Resistive Exercise (PRE) and Aerobic Exercise Compared with No Exercise for Adults Living with HIV

Outcomes	Anticipated absolute effects (95% CI)		Number of Participants (studies)	Quality of the evidence (GRADE)	Comments
	Risk with No Exercise	Risk with PRE or Combined PRE and Aerobic Exercise Compared with No Exercise			
**IMMUNOLOGICAL / VIROLOGICAL Viral Load (log10copies)**	The mean Viral Load (log10copies) in the control group was 0	The mean Viral Load (log10copies) in the intervention group was 0.12 log10copies higher (0.23 lower to 0.46 higher)	99 (4 RCTs)	⨁⨁⨁⊝ MODERATE	No significant difference in change in viral load between groups.
**CARDIORESPIRATORY VO2 Max (ml/kg/min)**	The mean VO2max (ml/kg/min) in the control group was 0	The mean VO2 max (ml/kg/min) in the intervention group was 3.71 ml/kg/min higher (1.73 higher to 5.70 higher)	82 (3 RCTs)	⨁⨁⨁⊝ MODERATE	***Significant improvement in VO2max among exercisers compared with non-exercisers.**<br>**^Estimate suggests a potential clinically important improvement in VO2max.**
**STRENGTH Upper Body - Chest Press (1 repetition maximum; kg)**	The mean Chest Press (1 repetition maximum; kg) in the control group was 0	The mean Chest Press (1 repetition maximum; kg) in the intervention group was 11.86 kg higher (2.37 higher to 21.36 higher)	44 (2 RCTs)	⨁⨁⊝⊝ LOW	***Significant improvement in upper body strength among exercisers compared with non-exercisers.**<br>**^Estimate suggests a potential clinically important improvement in strength.**

**STRENGTH**<br>**Lower Body - Leg Press (1 repetition maximum; kg)**	The mean Leg Press (1 repetition maximum; kg) in the control group was 0	The mean Leg Press (1 repetition maximum; kg) in the intervention group was 50.96 kg higher (13.01 lower to 114.92 higher)	44<br>(2 RCTs)	⊕⊝⊝⊝<br>VERY LOW	No significant difference between groups, but a trend towards an improvement in lower body strength among exercisers compared with non-exercisers.
**WEIGHT**<br>**Body Weight (kg)**	The mean Body Weight (kg) in the control group was 0	The mean Body Weight (kg) in the intervention group was 2.5 kg higher (0.32 higher to 4.67 higher)	129<br>(5 RCTs)	⊕⊝⊝⊝<br>VERY LOW	***Significant increase in body weight among exercisers compared with non-exercisers. Increase in body weight interpreted as a favourable outcome as reflection of increase in muscle mass.**
**BODY COMPOSITION**<br>**Body Mass Index (kg/m2)**	The mean Body Mass Index (kg/m^2) in the control group was 0	The mean Body Mass Index (kg/m2) in the intervention group was 0.40 kg/m^2 higher (0.22 lower to 1.03 higher)	131<br>(5 RCTs)	⊕⊕⊕⊝<br>MODERATE	No significant difference in change in body mass index between groups.
**BODY COMPOSITION**<br>**Fat Mass (kg)**	The mean Fat Mass (kg) in the control group was 0	The mean Fat Mass (kg) in the intervention group was 0.36 kg higher (0.5 lower to 1.23 higher)	103<br>(4 RCTs)	⊕⊕⊕⊝<br>MODERATE	No significant difference in change in fat mass between groups.

LEGEND: *Bold and asterisk text in the comments section indicates significant increase/improvement for the outcome. Increases in strength and body weight were considered favourable outcomes with exercise. ^Estimate suggests a potential clinically important change in outcome.

<u>GRADE Working Group grades of evidence</u>

High quality: We are very confident that the true effect lies close to that of the estimate of the effect.

Moderate quality: We are moderately confident in the effect estimate: The true effect is likely to be close to the estimate of the effect, but there is a possibility that it is substantially different.

Low quality: Our confidence in the effect estimate is limited: The true effect may be substantially different from the estimate of the effect.

Very low quality: We have very little confidence in the effect estimate: The true effect is likely to be substantially different from the estimate of effect.
